# Harmonization of Neuroticism and Extraversion phenotypes across inventories and cohorts in the Genetics of Personality Consortium: an application of Item Response Theory

**DOI:** 10.1007/s10519-014-9654-x

**Published:** 2014-05-15

**Authors:** Stéphanie M. van den Berg, Marleen H. M. de Moor, Matt McGue, Erik Pettersson, Antonio Terracciano, Karin J. H. Verweij, Najaf Amin, Jaime Derringer, Tõnu Esko, Gerard van Grootheest, Narelle K. Hansell, Jennifer Huffman, Bettina Konte, Jari Lahti, Michelle Luciano, Lindsay K. Matteson, Alexander Viktorin, Jasper Wouda, Arpana Agrawal, Jüri Allik, Laura Bierut, Ulla Broms, Harry Campbell, George Davey Smith, Johan G. Eriksson, Luigi Ferrucci, Barbera Franke, Jean-Paul Fox, Eco J. C. de Geus, Ina Giegling, Alan J. Gow, Richard Grucza, Annette M. Hartmann, Andrew C. Heath, Kauko Heikkilä, William G. Iacono, Joost Janzing, Markus Jokela, Lambertus Kiemeney, Terho Lehtimäki, Pamela A. F. Madden, Patrik K. E. Magnusson, Kate Northstone, Teresa Nutile, Klaasjan G. Ouwens, Aarno Palotie, Alison Pattie, Anu-Katriina Pesonen, Ozren Polasek, Lea Pulkkinen, Laura Pulkki-Råback, Olli T. Raitakari, Anu Realo, Richard J. Rose, Daniela Ruggiero, Ilkka Seppälä, Wendy S. Slutske, David C. Smyth, Rossella Sorice, John M. Starr, Angelina R. Sutin, Toshiko Tanaka, Josine Verhagen, Sita Vermeulen, Eero Vuoksimaa, Elisabeth Widen, Gonneke Willemsen, Margaret J. Wright, Lina Zgaga, Dan Rujescu, Andres Metspalu, James F. Wilson, Marina Ciullo, Caroline Hayward, Igor Rudan, Ian J. Deary, Katri Räikkönen, Alejandro Arias Vasquez, Paul T. Costa, Liisa Keltikangas-Järvinen, Cornelia M. van Duijn, Brenda W. J. H. Penninx, Robert F. Krueger, David M. Evans, Jaakko Kaprio, Nancy L. Pedersen, Nicholas G. Martin, Dorret I. Boomsma

**Affiliations:** 1 Department of Research Methodology, Measurement and Data-Analysis, University of Twente, Enschede, The Netherlands; 2 Department of Biological Psychology, VU University, Amsterdam, The Netherlands; 3 Department of Psychology, University of Minnesota, Elliott Hall, Minneapolis, MN USA; 4 Institute of Public Health, University of Southern Denmark, Odense, Denmark; 5 Department of Medical Epidemiology and Biostatistics, Karolinska Institutet, Stockholm, Sweden; 6 National Institute on Aging, NIH, Baltimore, MD USA; 7 College of Medicine, Florida State University, Tallahassee, FL USA; 8 QIMR Berghofer Medical Research Institute, Brisbane, QLD Australia; 9 Department of Developmental Psychology and EMGO Institute for Health and Care Research, VU University Amsterdam, Amsterdam, The Netherlands; 10 Department of Epidemiology, Erasmus University Medical Center, Rotterdam, The Netherlands; 11 Department of Psychology, University of Illinois at Urbana-Champaign, Champaign, IL USA; 12 Estonian Genome Center, University of Tartu, Tartu, Estonia; 13 Department of Psychiatry, EMGO+ Institute, Neuroscience Campus Amsterdam, VU University Medical Center Amsterdam, Amsterdam, The Netherlands; 14 MRC Human Genetics, MRC IGMM, Western General Hospital, University of Edinburgh, Edinburgh, Scotland, UK; 15 Department of Psychiatry, University of Halle, Halle, Germany; 16 Institute of Behavioural Sciences, University of Helsinki, Helsinki, Finland; 17 Folkhälsan Research Center, Helsinki, Finland; 18 Department of Psychology, University of Edinburgh, Edinburgh, UK; 19 Centre for Cognitive Ageing and Cognitive Epidemiology, University of Edinburgh, Edinburgh, UK; 20 Department of Psychiatry, Washington University School of Medicine, St. Louis, MO USA; 21 Department of Psychology, University of Tartu, Tartu, Estonia; 22 Estonian Academy of Sciences, Tallinn, Estonia; 23 Department of Public Health, Hjelt Institute, University of Helsinki, Helsinki, Finland; 24 National Institute for Health and Welfare (THL), Helsinki, Finland; 25 Centre for Population Health Sciences, Medical School, University of Edinburgh, Edinburgh, UK; 26 MRC Integrative Epidemiology Unit, School of Social and Community Medicine, University of Bristol, Bristol, UK; 27 Department of General Practice and Primary Health Care, University of Helsinki, Helsinki, Finland; 28 Unit of General Practice, Helsinki University Central Hospital, Helsinki, Finland; 29 Vasa Central Hospital, Vaasa, Finland; 30 Donders Institute for Cognitive Neuroscience, Radboud University Nijmegen, Nijmegen, The Netherlands; 31 Department of Psychiatry, Radboud University Nijmegen Medical Center, Nijmegen, The Netherlands; 32 Department of Human Genetics, Radboud University Nijmegen Medical Center, Nijmegen, The Netherlands; 33 Department of Psychology, School of Life Sciences, Heriot-Watt University, Edinburgh, UK; 34 Department of Clinical Chemistry, Fimlab Laboratories and School of Medicine, University of Tampere, Tampere, Finland; 35 Department of Health Evidence, Radboud University Nijmegen Medical Center, Nijmegen, The Netherlands; 36 Department of Urology, Radboud University Nijmegen Medical Center, Nijmegen, The Netherlands; 37 Institute of Genetics and Biophysics “A. Buzzati-Traverso” – CNR, Naples, Italy; 38 Wellcome Trust Sanger Institute, Wellcome Trust Genome Campus, Hinxton, Cambridge, UK; 39 Institute for Molecular Medicine Finland (FIMM), University of Helsinki, Helsinki, Finland; 40 Department of Public Health, Faculty of Medicine, University of Split, Split, Croatia; 41 Department of Psychology, University of Jyväskylä, Jyväskylä, Finland; 42 Department of Clinical Physiology and Nuclear Medicine, Turku University Hospital, Turku, Finland; 43 Research Centre of Applied and Preventive Cardiovascular Medicine, University of Turku, Turku, Finland; 44 Department of Psychological & Brain Sciences, Indiana University, Bloomington, IN USA; 45 Department of Psychological Sciences and Missouri Alcoholism Research Center, University of Missouri, Columbia, MO USA; 46 Department of Psychological Methods, University of Amsterdam, Amsterdam, The Netherlands; 47 Department of Psychiatry, University of California, La Jolla, CA, USA; 48 Department of Public Health and Primary Care, Trinity College Dublin, Dublin, Ireland; 49 Department of Cognitive Neuroscience, Radboud University Nijmegen Medical Center, Nijmegen, The Netherlands; 50 Behavioral Medicine Research Center, Duke University School of Medicine, Durham, NC USA; 51 Department of Behavioural Sciences, OMD, University of Twente, PO Box 217, 7500 AE Enschede, The Netherlands

**Keywords:** Personality, Item-Response Theory, Measurement, Genome-wide association studies, Consortium, Meta-analysis

## Abstract

Mega- or meta-analytic studies (e.g. genome-wide association studies) are increasingly used in behavior genetics. An issue in such studies is that phenotypes are often measured by different instruments across study cohorts, requiring harmonization of measures so that more powerful fixed effect meta-analyses can be employed. Within the Genetics of Personality Consortium, we demonstrate for two clinically relevant personality traits, Neuroticism and Extraversion, how Item-Response Theory (IRT) can be applied to map item data from different inventories to the same underlying constructs. Personality item data were analyzed in >160,000 individuals from 23 cohorts across Europe, USA and Australia in which Neuroticism and Extraversion were assessed by nine different personality inventories. Results showed that harmonization was very successful for most personality inventories and moderately successful for some. Neuroticism and Extraversion inventories were largely measurement invariant across cohorts, in particular when comparing cohorts from countries where the same language is spoken. The IRT-based scores for Neuroticism and Extraversion were heritable (48 and 49 %, respectively, based on a meta-analysis of six twin cohorts, total N = 29,496 and 29,501 twin pairs, respectively) with a significant part of the heritability due to non-additive genetic factors. For Extraversion, these genetic factors qualitatively differ across sexes. We showed that our IRT method can lead to a large increase in sample size and therefore statistical power. The IRT approach may be applied to any mega- or meta-analytic study in which item-based behavioral measures need to be harmonized.

**Electronic supplementary material:**

The online version of this article (doi:10.1007/s10519-014-9654-x) contains supplementary material, which is available to authorized users.

## Introduction

Mega- or meta-analytic studies (e.g. genome-wide association (GWA) studies) are increasingly used in behavior genetics. Because phenotypes have not always been assessed similarly across cohorts (and sometimes not even within cohorts), measures need to be harmonized, that is, phenotypic scores need to be made comparable such that data from individuals who were assessed by different inventories can be compared meaningfully. Such harmonization then enables fixed effect meta-analytic analyses (Hedges and Vevea 1998). Meta-analytic studies are required when effect sizes are small such as for complex human traits. For example, GWA studies for psychiatric disorders have led to important discoveries, but for many disorders, individual variants typically explain less than 1 % of the heritability, although in unison they can explain quite a large proportion of phenotypic variation (Craddock et al. 2008; Lee et al. 2013; Ripke et al. 2013; Sullivan et al. 2012). Sample size determines the number of significant loci discovered (Sullivan et al. 2012), so that meta-analysis of results is the gold standard. Consortium GWA studies for traits such as height and body-mass index now report sample sizes of >100,000 (Berndt et al. 2013; Lango Allen and et al. 2010; Speliotes et al. 2010). Consortia for psychiatric disorders and behavioral traits have also been formed, with sample sizes increasing rapidly to hundreds of thousands (Rietveld et al. 2012; Ripke et al. 2011; Wray et al. 2012), leading to the discovery of novel loci for psychiatric disorders and educational attainment. Thus, large sample sizes are essential for behavioral phenotypes.

A meta-analysis of behavioral measures will have most power if the same reliable and valid measurement instrument is administered in all cohorts. In practice, however, different instruments are often used, and, even when the instrument is the same, translations into different languages may cause problems. To tackle the problem that different inventories may not assess the same phenotype, we demonstrate how Item-Response Theory (IRT) test linking can be applied to map item data from different inventories to a common metric. We conduct such an analysis for Neuroticism and Extraversion personality traits, based on data from the Genetics of Personality Consortium (GPC). If different inventories indeed measure the same phenotype, the only requirement for this approach is that multiple inventories have been administered in at least a subset of individuals. That is, in order to be able to harmonize across different inventories, some participants must have filled in multiple inventories so that they can function as a “bridge” between inventories. This can be done if we assume that the true phenotype (personality) does not change between the multiple assessments. If this can be assumed, then for all individuals in the different (sub-)cohorts, a score on the latent construct can be estimated based on all available item data for that person. The IRT-based score estimates for Neuroticism and Extraversion can subsequently be meta-analyzed to assess heritability, or can be used as phenotypes in GWA or brain-imaging studies.

This IRT approach has multiple advantages. First, within each cohort there is increased measurement reliability, because when multiple inventories have been administered to the same individual, scores can be estimated using the items from all relevant inventories. In addition, items can be differentially and optimally weighted if necessary, and items that do not fit the measurement model can be identified and omitted, thereby increasing power. Subgroups of individuals that were assessed with only a subset of items can now also be included in the study. Moreover, the IRT approach can statistically evaluate the extent to which different inventories actually measure the same construct. Lastly, IRT enables researchers to determine the extent of measurement invariance across cohorts: can scores across cohorts be quantitatively compared and therefore pooled and meaningfully used in a meta-analysis?

Applying the IRT method to Neuroticism and Extraversion is especially relevant for the field of behavior genetics, as these personality traits are correlated with numerous other traits and disorders, not only phenotypically but also genetically (Heath et al. 1994; Hopwood et al. 2011; Klein et al. 2011; Markon et al. 2005; Samuel and Widiger 2008). For example, Neuroticism is highly related to a variety of psychiatric disorders, including major depression and borderline personality disorder (Distel et al. 2009; Kendler and Myers 2009), and Extraversion is associated with alcohol use (Dick et al. 2013). Earlier GWA studies of personality (De Moor et al. 2010; Service et al. 2012; Shifman et al. 2008; Terracciano et al. 2010; van den Oord et al. 2008) focused on single inventories, hence hampering sample size, and few, if any, genome-wide significant loci were detected. Large sample sizes are needed, which can be achieved by pooling results from multiple inventories.

This study included data obtained from 160,958 individuals from 23 cohorts, of which 6 were twin cohorts. Neuroticism and Extraversion were assessed by 9 different personality inventories; 7 cohorts assessed more than one inventory. The first objective was to determine the feasibility of the IRT approach in linking Neuroticism and Extraversion item data from different inventories: to what extent do the different inventories measure the same constructs? For instance, Harm Avoidance correlates moderately high with Neuroticism (r = 0.5–0.6) (De Fruyt et al. 2000). Therefore, we expect that mapping item data from Harm Avoidance with Neuroticism will be less perfect than mapping Neuroticism item data from other personality inventories (e.g. EPQ versus NEO neuroticism). We expect that this is even more the case for mapping Reward Dependence with Extraversion. Here we determine to what extent cross-inventory mapping is feasible, for the purpose of a GWAS meta-analysis in mind. The second objective was to test for measurement invariance across cohorts, and the third objective was to establish the heritability of the harmonized Neuroticism and Extraversion scores in the six participating twin cohorts. Sex differences in the genetic background of Neuroticism and Extraversion were studied, as well as the contribution of non-additive genetic factors. The contribution of non-additive genetic factors to variation in personality traits has been extensively discussed in the literature (Keller et al. 2005), but their assessment requires a large sample (Posthuma and Boomsma 2000). Lastly, we studied the theoretical increase in power of finding a quantitative trait locus due to the harmonization of phenotypes in two large cohorts.

## Materials and methods

### Cohorts

Twenty-three cohorts of the GPC were included in this study (for detailed descriptions, see Supplementary Materials Online). Seventeen cohorts originated from Europe, 4 cohorts were from the USA and 2 cohorts from Australia. Most cohorts are large epidemiological studies. Some of the cohorts focused on specific birth cohorts and/or recruited individuals of specific regions in the country (e.g. ERF, VIS, KORCULA, NBS, LBC1921, LBC1936 and HBCS), or targeted twins and their family members (QIMR cohorts, NTR, MCTFR, STR, Finnish Twin Cohort). Three cohorts were designed to include cases and controls for Nicotine dependence, Alcoholism or Mood and Anxiety disorders (respectively, COGEND, SAGE-COGA and NESDA). The data collection in some of the cohorts is longitudinal in nature.

### Personality assessment

Supplementary Table 1 and Supplementary Fig. 3 give an overview of the personality inventories administered in each cohort. The Supplementary Materials Online describes these inventories in detail. For the Neuroticism analysis, we included all Neuroticism items from the NEO, the International Personality Item Pool (IPIP) and Eysenck (EPQ, EPI, ABV) inventories, the Harm Avoidance (HA) items from the Temperament and Character Inventory (TCI), and the Negative Emotionality (NEM) items (excluding the aggression items) from the Multidimensional Personality Questionnaire (MPQ). The Neuroticism scales of the NEO, IPIP and Eysenck inventories consist of different items, but there is strong overlap in item content and the sum scores correlate highly across inventories (Aluja et al. 2004; Draycott and Kline 1995; Larstone et al. 2002). HA correlates most strongly with Neuroticism (as assessed with the NEO-PI-R or EPQ-R) (De Fruyt et al. 2000; Gillespie et al. 2001). NEM corresponds most closely to Neuroticism, although NEM is a broader concept because it also includes items about aggressive behavior.

For the Extraversion analysis, all Extraversion items from the NEO, IPIP and Eysenck inventories were analyzed, a selection of Reward Dependence (RD) items from the TCI, and the Positive Emotionality (PEM) items from the MPQ. Extraversion sum scores derived from the NEO, IPIP and Eysenck inventories correlate highly across inventories (Aluja et al. 2004; Draycott and Kline 1995; Larstone et al. 2002). The relationship between Extraversion and the temperament traits is less clear, but Extraversion correlates strongest with RD (De Fruyt et al. 2000; Gillespie et al. 2001). Based on the item correlations among the RD items with the Extraversion items from the NEO-PI-R and EPQ in the HBCS, PAGES and QIMR adults cohorts, we decided to include a subset of RD items that correlated strongest with the Extraversion items (see Supplementary Fig. 3 for number of items included and Supplementary Table 2 for overview of the items).

### Estimating Neuroticism and Extraversion scores

The harmonization goal is to estimate personality scores that are not biased by the number of items and the specific inventory used. In the field of IRT, such harmonization is termed ‘test linking’. By fitting IRT models (Lord 1980) to item data, personality scores can be estimated conditional on the observed items and their respective item parameters. This leads to personality scores for individuals that are comparable irrespective of what items were assessed in a particular individual. For example, image an intelligence assessment: If we know that items 1–10 are very easy test items, and items 11–20 are very difficult, we are pretty confident that a person that scores 1 on the items 1–10 is less bright than a person that scores 9 on items 11–20. The exact knowledge of the difficulties of the 20 items allows us to estimate the difference in intelligence.

A basic IRT model assumes a one-dimensional latent variable representing the trait that predicts the probability of a certain response on a particular item: the higher the latent trait value, the higher the probability of a high score on the item. Item parameters determine the exact relationship between the latent trait and the probability of the response to a particular item. The so-called difficulty parameter provides information about the general probability of a positive response to a particular item, and is very similar to the threshold parameter in liability models. The discrimination parameter value of an item indicates how strong the relationship is between the latent trait and the item response variable, and is therefore similar to a factor loading. Because latent scores are estimated conditional on the item parameters for the administered items, the scoring process becomes independent of the particular items in the test. For example, this allows the comparison of a child’s achievement on a test with easy questions with the achievement of another child on a test with difficult questions. IRT test linking was applied in each cohort separately and used to link all data from one cohort to one common metric for Neuroticism and one common metric for Extraversion. For more details, see Supplementary Materials Online.

### Appropriateness of Item Response Theory to harmonize Neuroticism and Extraversion scores

We assessed whether the IRT Neuroticism and Extraversion scores in the 23 cohorts were truly independent of the specific inventory used. First, the appropriateness of linking tests *within cohorts* was investigated by testing basic assumptions of IRT models: the idea that scoring is independent of the specific item set that was administered (local independence), and unidimensionality. For every cohort and every inventory separately, item parameters were estimated based on data from individuals without missing data. Such a set of parameter values for a particular sample of items assessed in a particular sample is termed a calibration. Calibrations were also obtained for *combinations* of item sets from various inventories, if there was a subsample of individuals that was assessed with those inventories. Based on these calibrations, (i.e., sets of item parameter values), latent scores can be estimated for those individuals for which one has either complete data or data with some missing values, assuming these are missing at random. In order to investigate local independence, latent scores for a particular item set (say, item scores for NEO-PI-R) were estimated and compared based on different calibrations: one based on the calibration of several inventories combined (e.g., NEO-PI-R and EPQ-R Neuroticism) and one based on only one inventory (NEO-PI-R items). The resulting scores were then correlated. A correlation of 1 indicates that the estimated scores are completely independent of what inventory was used for assessment (see also Supplementary Materials Online).

Unidimensionality was assessed by plotting the test information curves (TICs) (Lord 1980; van den Berg and Service 2012) for inventories separately and with two or more inventories combined. If two tests measure the same underlying construct, the TIC of the tests combined should be the sum of the TICs of the two separate tests. These curves also show the increase in measurement precision for those individuals that were administered multiple inventories.

The choice for the above approach to assessing model fit, which is a bit unconventional, was motivated by the fact that the personality inventories are well-developed and validated instruments. Also, from previous research we know that two-parameter models generally are more appropriate for personality data than one- and three-parameter models (Chernyshenko et al. 2001; Reise and Waller 1990). As one aim is to use as much information as possible from the personality inventories, to establish a linear relationship between personality scales and an external variable, such as a SNP, we chose to retain all items in the analyses.

The above analysis determines whether within cohorts, items from inventories can be combined, that is, whether different inventories can be used to measure the same trait. In addition, it is important to assess whether *across cohorts*, the same trait is being measured. If Neuroticism and Extraversion were very differently expressed across cohorts, a meta-analysis is rather meaningless. Due to a host of reasons (culture, language, sample selection criteria, etc.), the same test items might have different parameters across cohorts. Ignoring these differences results in systematic bias when comparing individual sum scores from different cohorts. The assumption of equal item parameters across groups is usually termed measurement invariance (Meredith 1993). If one item has different parameter values across groups, this is called differential item functioning (DIF) (Glas 1998, 2001; Speliotes et al. 2010). There are two ways of dealing with DIF, either (1) omitting the item entirely in estimating individual scores, or (2) allowing for different item parameters for that particular DIF item across groups (Weisscher et al. 2010). The first approach leads to loss of information, so that the second is generally more attractive.

A new alternative Bayesian method for modeling measurement non-invariance (Verhagen and Fox 2013a, b) was applied to assess variance of item parameters across cohorts and that identifies true differences in means and variances of Neuroticism and Extraversion across cohorts, while controlling for any measurement non-invariance. The Bayesian approach allows for estimating complicated models in a straightforward way, and through hierarchical modeling one borrows statistical strength for small cohorts from information in larger cohorts. The Bayesian hierarchical approach assumes there is at least some violation of measurement invariance, and quantifies its extent. Since there are some important differences across cohorts in terms of population and language, we expect there will be at least some difference in item parameters across cohorts.

**Fig. 1:**
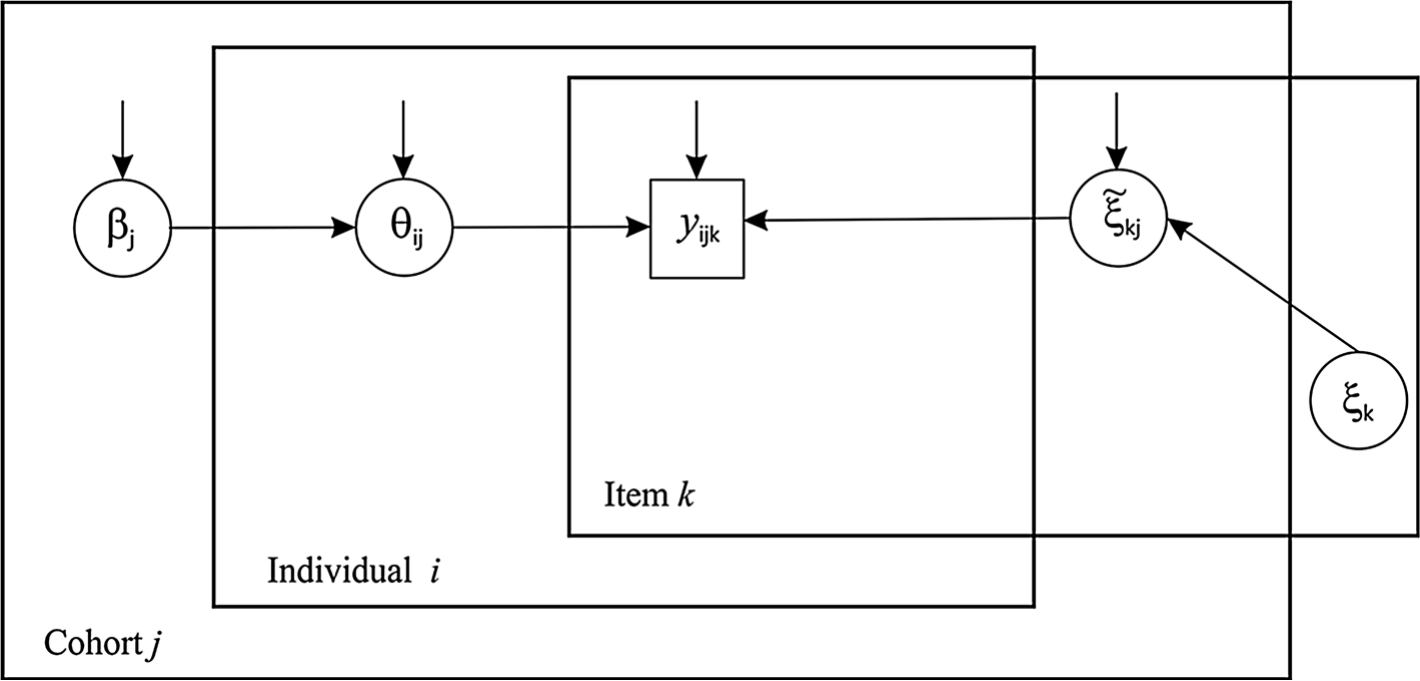
A graph representation of the hierarchical model for measurement variance. Item parameters *ξ* (thresholds and discrimination parameter) are allowed to vary across cohorts, but person parameters are allowed to vary both across cohorts and within cohorts. Observed response *Y*_*ijk*_ from person *i* in cohort *j* to item *k* is predicted by a latent score *θ*_*ij*_ for that person and item parameters *ξ*_*kj*_ for item *k* that is specific for cohort *j*

In the Bayesian hierarchical approach, item and person parameters are estimated using a Markov Chain Monte Carlo procedure, in which cohort-specific item parameters are considered level-1 parameters randomly distributed around overall mean item parameters at level 2. See Fig. 1 for a graph representation of the hierarchical structure of both item and person parameters across cohorts. As the identification constraint, the average difficulty of the items is assumed equal across cohorts. That is, cohorts may differ in mean and variance of the latent trait, and particular item parameters might be different across cohorts, but the *average* difficulty of items is the same (for example, in case of an IQ test for males and females: the assumption is that overall the test has the same difficulty, although it can be the case that some items are relatively more difficult for males, and other items are relatively more difficult for females). In addition, to identify the variance of the scale the product of the discrimination parameters was fixed at 1. Allowing for such random fluctuations in difficulty and discrimination across cohorts is also referred to as the assumption of approximate measurement invariance. This Bayesian method was only applied to NEO-FFI and EPQ-R test items, as for those tests, the numbers of cohorts were sufficiently large. We randomly selected 1,000 individuals from each cohort (or all individuals if sample size was smaller) and determined which items showed considerable DIF across cohorts by computing Bayes factors (Verhagen and Fox 2013a, b). When testing invariance hypotheses, an advantage of the Bayes factor is that you can gather evidence in favor of the (null) hypothesis of invariance. A Bayes factor smaller than 0.3 was regarded as clear evidence of DIF. A Bayes factor larger than 3 was regarded as evidence of measurement invariance (i.e., no DIF). Taking into account possible DIF, all individuals with either NEO or EPQ data were mapped to a common scale for Neuroticism and Extraversion and mean Neuroticism and Extraversion scores and variances were estimated for each cohort.

Significant DIF does not imply that its effects are dramatic. To assess the extent to which DIF results in different scoring, depending on what calibration is used, Neuroticism and Extraversion scores were estimated using different cohort-specific calibrations and these were compared. For example, how much would the estimated scores for individuals in the Dutch NTR sample differ if instead of using the NTR calibration (i.e., using item parameters as estimated using NTR data), the Finnish HBCS calibration were used? If measurement invariance holds perfectly, the correlation between the different score estimates should be very close to 1. These correlations were computed for NEO-FFI, NEO-PI-R and EPQ inventories in the appropriate cohorts.

### Meta-analysis of heritability

In each of the 6 cohorts with twin data separately, twin correlations for the IRT latent trait scores were estimated using the structural equation modeling package OpenMx within the statistical software program R (Boker et al. 2011). This was done by fitting a fully saturated model using full information likelihood to the data of twins in five sex-by-zygosity groups: monozygotic male twin pairs (MZM), dizygotic male twin pairs (DZM), monozygotic female twin pairs (MZM), dizygotic female twin pairs (DZM) and dizygotic twin pairs of opposite sex (DOS; if available in the particular cohort). Twin pairs in which Neuroticism and Extraversion scores were available for both twins were included, as well as twin pairs for which information was available for only one of the twins. In each cohort including a DOS group, 16 parameters were estimated: 5 means (5 sex by zygosity groups), 1 regression parameter for the effect of age on the means, 5 variances (5 sex by zygosity groups) and 5 covariances (for 5 sex by zygosity groups). In the cohorts without a DOS group, 4 means, 1 regression parameter for age, 4 variances and 4 covariances were estimated (13 parameters in total). The 4 or 5 covariances were standardized in each sex-by-zygosity group in order to obtain 4 or 5 twin correlations in each cohort. In addition, the 95 % confidence intervals for the twin correlations were computed. It was further tested whether the twin correlations could be constrained to be equal across sex (MZM = MZF and DZM = DZF = DOS).

Under the classical twin model assumptions, the expected MZ twin correlation is a function of the proportions of variance in a trait explained by additive (*h* ^2^) and non-additive (*d* ^2^) genetic effects: r(MZ) = *h* ^2^ + *d* ^2^. The expected DZ twin correlation is a different function of these two types of effects: r(DZ) = ½*h* ^2^ + ¼*d* ^2^. IRT-score-based twin correlations (Table 1) were used as the basis to assess both qualitative and quantitative sex effects. This was done by fitting the same model to data from all six cohorts simultaneously allowing for different estimates of *h* ^2^ and *d* ^2^ in each sex, and allowing the opposite-sex twin correlation to be different from its expectation, ½*h*_*m*_ *h*_*f*_ + ¼ *d*_*m*_ *d*_*f*_. The estimates of parameters (*h* ^2^, *e* ^2^ and *d* ^2^ by sex) thus were constrained to be the same across cohorts. First it was tested whether the correlation in opposite-sex twins could be equated to the expectation above (i.e. testing for qualitative sex effects). Next, it was tested whether the relative sizes of the genetic components could be equated across sexes, that is, whether *h*_*m*_^2^ = *h*_*f*_^2^ and *d*_*m*_^2^ = *d*_*f*_^2^. Lastly, it was tested whether non-additive genetic effects were present, by comparing the fit of the model with a model in which *d* ^2^ = 0.

**Table 1:** Twin correlations for the IRT-based Neuroticism and Extraversion scores

Cohort	Twin pairs	Trait	r_MZ_	N	95 % CI	r_DZ_	N	95 % CI
7. FINNISH TWINS	M–M	Neuroticism	0.43	1998	0.39–0.47	0.20	4862	0.16–0.23
		Extraversion	0.44	1999	0.40–0.48	0.14	4861	0.11–0.17
	F–F	Neuroticism	0.48	2226	0.45–0.52	0.19	4658	0.16–0.22
		Extraversion	0.52	2227	0.49–0.55	0.15	4663	0.12–0.18
	All	Neuroticism	0.46	4224	0.43–0.48	0.19	9520	0.17–0.21
		Extraversion	0.48	4226	0.46–0.51	0.14	9524	0.12–0.17
12. MCTFR	M–M	Neuroticism	0.53	922	0.47–0.60	0.17	506	0.05–0.28
		Extraversion	0.52	922	0.45–0.58	0.23	506	0.11–0.34
	F–F	Neuroticism	0.45	1054	0.38–0.52	0.26	580	0.15–0.37
		Extraversion	0.51	1054	0.45–0.57	0.13	580	0.02–0.25
	All	Neuroticism	0.48	1976	0.44–0.53	0.22	1086	0.14–0.30
		Extraversion	0.52	1976	0.47–0.56	0.17	1086	0.09–0.25
15. NTR	M–M	Neuroticism	0.45	1124	0.40–0.50	0.22	855	0.14–0.29
		Extraversion	0.47	1123	0.42–0.52	0.13	855	0.06–0.21
	F–F	Neuroticism	0.51	2249	0.47–0.54	0.23	1391	0.17–0.28
		Extraversion	0.49	2248	0.46–0.52	0.20	1392	0.14–0.26
	M–F	Neuroticism	–	–	–	0.21	2044	0.16–0.26
		Extraversion	–	–	–	0.14	2044	0.09–0.19
	All	Neuroticism	0.49	3373	0.46–0.52	0.22	4290	0.18–0.25
		Extraversion	0.48	3371	0.46–0.51	0.16	4291	0.13–0.19
18. QIMR adolescents	M–M	Neuroticism	0.51	304	0.42–0.59	0.27	252	0.15–0.38
		Extraversion	0.49	304	0.40–0.57	0.18	252	0.06–0.30
	F–F	Neuroticism	0.39	329	0.29–0.48	0.19	268	0.07–0.30
		Extraversion	0.45	329	0.36–0.53	0.19	268	0.07–0.31
	M–F	Neuroticism	–	–	–	0.21	463	0.13–0.30
		Extraversion	–	–	–	0.12	463	0.03–0.21
	All	Neuroticism	0.44	633	0.38–0.50	0.22	983	0.16–0.28
		Extraversion	0.47	633	0.40–0.53	0.16	983	0.09–0.22
19. QIMR adults	M–M	Neuroticism	0.45	1182	0.40–0.50	0.11	889	0.04–0.19
		Extraversion	0.48	1182	0.43–0.53	0.19	889	0.11–0.26
	F–F	Neuroticism	0.48	2075	0.45–0.52	0.22	1435	0.17–0.28
		Extraversion	0.48	2075	0.44–0.51	0.16	1435	0.11–0.21
	M–F	Neuroticism	–	–	–	0.13	1827	0.08–0.18
		Extraversion	–	–	–	0.14	1827	0.09–0.19
	All	Neuroticism	0.47	3257	0.44–0.50	0.16	4151	0.13–0.19
		Extraversion	0.48	3257	0.45–0.51	0.16	4151	0.12–0.19
21. STR	M–M	Neuroticism	0.54	3188	0.51–0.56	0.18	4841	0.15–0.21
		Extraversion	0.54	3188	0.51–0.56	0.25	4841	0.22–0.28
	F–F	Neuroticism	0.45	2830	0.42–0.49	0.16	4625	0.13–0.19
		Extraversion	0.44	2830	0.41–0.48	0.20	4625	0.17–0.23
	All	Neuroticism	0.51	6018	0.49–0.53	0.19	9466	0.17–0.21
		Extraversion	0.52	6018	0.50–0.54	0.26	9466	0.23–0.28

*r*_*MZ*_ correlation in monozygotic twin pairs, *r*_*DZ*_ correlation in dizygotic twin pairs, *N* number of twin pairs (pairs are included with personality data for both twins and with data for one twin), *95* *% CI* 95 % confidence interval, *M–M* male–male twin pairs,* F*–*F* female–female twin pairs, *M–F* male–female twin pairs, *All* twin pairs combined across gender

### Power study

For the NTR and the QIMR-adult cohorts, the increase in statistical power for a GWAS on Neuroticism was determined that results from the increase in sample size and measurement precision due to the IRT test linking. A baseline condition of using 12 NEO-FFI items as in a previous meta-analysis (De Moor et al. 2010) was compared with using all available data from NEO-PI-R and other available inventories. We assumed that genotype data was non-missing for all phenotypes. Power was computed for a single nucleotide polymorphism (SNP) explaining 0.1 % of true phenotypic variance (latent trait) with allele frequency 0.5. Item data were simulated with parameter settings equal to the observed parameter estimates in the empirical data. Sample sizes were also the same as in the empirical data. For each power estimate, 100 data sets were simulated and analyzed, and the proportion of *p*-values smaller than 10^−8^ was calculated.

## Results

### Estimating Neuroticism and Extraversion scores

Personality scores were estimated for 160,671 (Neuroticism) and 160,713 individuals (Extraversion). Correlations between estimated latent scores and sum scores were high for Neuroticism (79 % of the correlations >0.90, and 50 % >0.95; lowest correlation 0.73) and moderately high for Extraversion (82 % of the correlations >0.80, and 48 % >0.90; lowest correlation 0.60) (Table 2). Correlations were highest with NEO, EPQ and IPIP-based sum scores, and lowest with TCI-based sum scores.

**Table 2:** Correlations between the IRT-based Neuroticism and Extraversion scores and the personality inventory-based sum scores

	Neuroticism		Extraversion	
Cohort	**N**	**r**	**N**	**r**
1. ALSPAC	6,068	0.98 (IPIP)	6,072	0.97 (IPIP)
2. BLSA	1,917	0.96 (NEO-PI-R)	1,917	0.97 (NEO-PI-R)
3. CILENTO	800	0.97 (NEO-PI-R)	800	0.98 (NEO-PI-R)
4. COGEND	2,712	0.98 (NEO-FFI)	2,712	0.98 (NEO-FFI)
5. EGCUT	1,730	0.98 (NEO-PI-3)	1,730	0.98 (NEO-PI-3)
6. ERF	2,474	0.93 (NEO-FFI)	2,479	0.87 (NEO-FFI)
7. FINNISH TWINS	30,073	0.96 (NEO-FFI) 0.98 (EPI)	30,120	0.94 (NEO-FFI) 0.97 (EPI)
8. HBCS	1,698	0.91 (NEO-PI-R) 0.85 (TCI)	1,698	0.92 (NEO-PI-R) 0.63 (TCI)
9. KORCULA	810	0.97 (EPQ)	809	0.79 (EPQ)
10. LBC1921	478	0.96 (IPIP)	478	0.98 (IPIP)
11. LBC1936	1,032	0.92 (NEO-FFI) 0.92 (IPIP)	1,032	0.85 (NEO-FFI) 0.93 (IPIP)
12. MCTFR	9,063	0.97 (MPQ)	9,063	0.96 (MPQ)
13. NBS	1,818	0.96 (EPQ)	1,821	0.96 (EPQ)
14. NESDA	2,961	0.99 (NEO-FFI)	2,961	0.96 (NEO-FFI)
15. NTR	31,299	0.91 (NEO-FFI) 0.89 (ABV)	31,294	0.85 (NEO-FFI) 0.86 (ABV)
16. ORCADES	602	0.98 (EPQ)	602	0.88 (EPQ)
17. PAGES	476	0.95 (NEO-PI-R) 0.73 (TCI)	476	0.93 (NEO-PI-R) 0.60 (TCI)
18. QIMR-adolescents	4,100	0.93 (NEO-PI-R) 0.94 (NEO-FFI) 0.86 (JEPQ)	4,100	0.88 (NEO-PI-R) 0.77 (NEO-FFI) 0.81 (JEPQ)
19. QIMR-adults	26,681	0.94 (NEO-PI-R) 0.92 (NEO-FFI) 0.86 (EPQ) 0.88 (TCI) 0.87 (MPQ)	26,681	0.90 (NEO-PI-R) 0.89 (NEO-FFI) 0.94 (EPQ) 0.64 (TCI) 0.85 (MPQ)
20. SAGE-COGA	649	0.97 (TCI)	649	0.89 (TCI)
21. STR	30,264	0.96 (EPI)	30,253	0.97 (EPI)
22. VIS	909	0.98 (EPQ)	909	0.75 (EPQ)
23. YOUNG FINNS	2,057	0.97 (NEO-FFI)	2,057	0.96 (NEO-FFI)
TOTAL	160,671		160,713	

### Appropriateness of Item Response Theory to harmonize Neuroticism and Extraversion scores

To assess whether test linking was successful within the seven cohorts that assessed more than one personality inventory, latent scores were computed based on different calibrations. In the majority of cohorts, the correlations among estimated scores were very high for most of the inventories (r > 0.96). Only for TCI Neuroticism in the HBCS cohort, was the correlation lower (r = 0.87). Thus, the latent scores are largely independent of the inventories included. TICs for these cohorts are presented in Supplementary Figs. 4–27. Supplementary Figs. 11, 14, 18, and 20 thru 23 show that combining tests always leads to higher information content, and therefore more measurement precision for those individuals with multiple-inventory data. However, the TICs of the combined tests are not a simple sum of the TICs of the individual tests, showing that the personality inventories largely, but not completely, measure the same phenotypes.

To assess whether personality scores could be compared *across* cohorts, latent scores in each cohort were estimated several times based on different values for the item parameters coming from different cohorts (different calibrations). That is, a certain pattern of item responses was used to estimate the latent trait based on the item parameters as calibrated in one cohort, and this was repeated but then using item parameters as calibrated in another cohort. The correlations (see Supplementary Tables 4 and 5) are generally very high (most >0.95; only 3 out of the 84 < 0.90, with the lowest correlation 0.81). Thus, ranking is not much affected by the particular cohort that individuals were in.

**Fig. 2:**
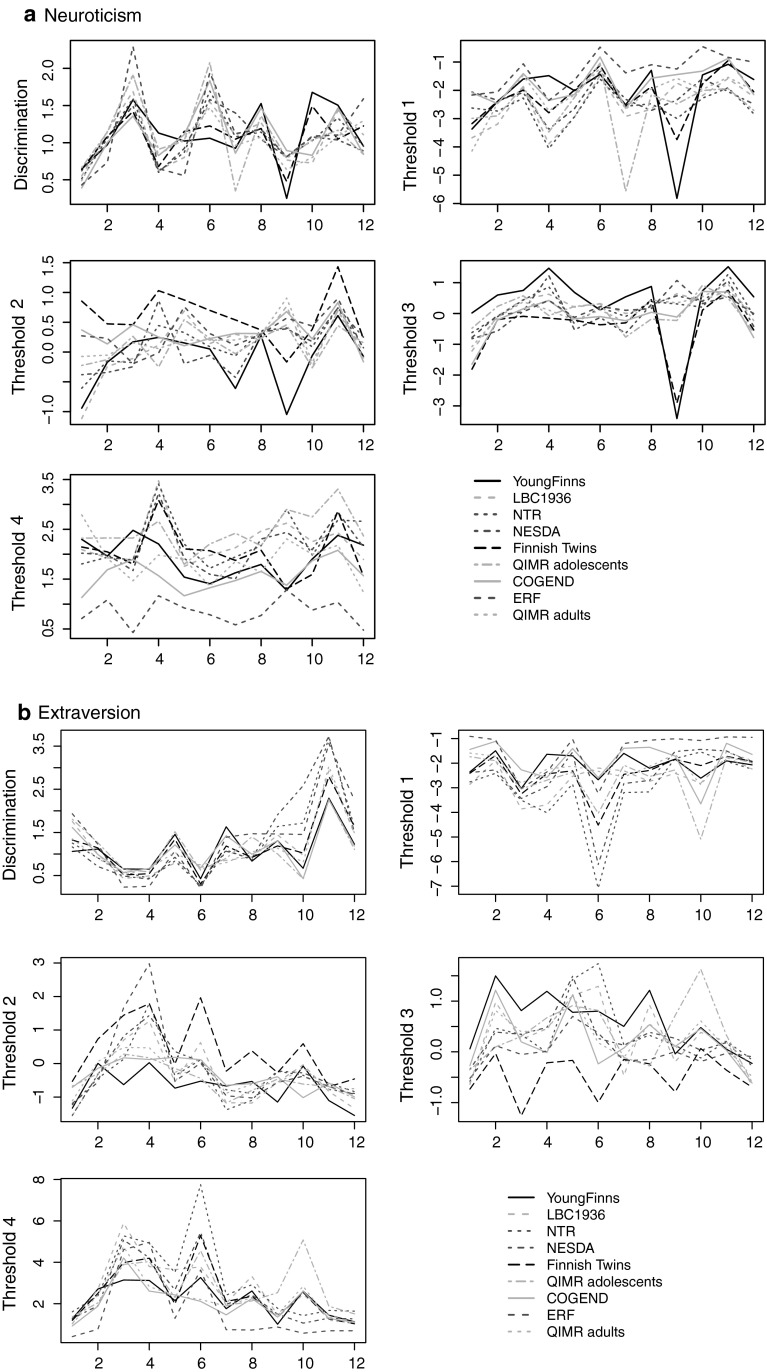
Parameter estimates (thresholds and discrimination parameters) for 12 items (*x*-axis) from the NEO-FFI personality inventory for different cohorts, separately for Neuroticism and Extraversion. In *black*, the item parameter values for Finnish language cohorts, in *green* for Dutch language cohorts, and in *red* for English language cohorts (Color figure online)

**Fig. 3:**
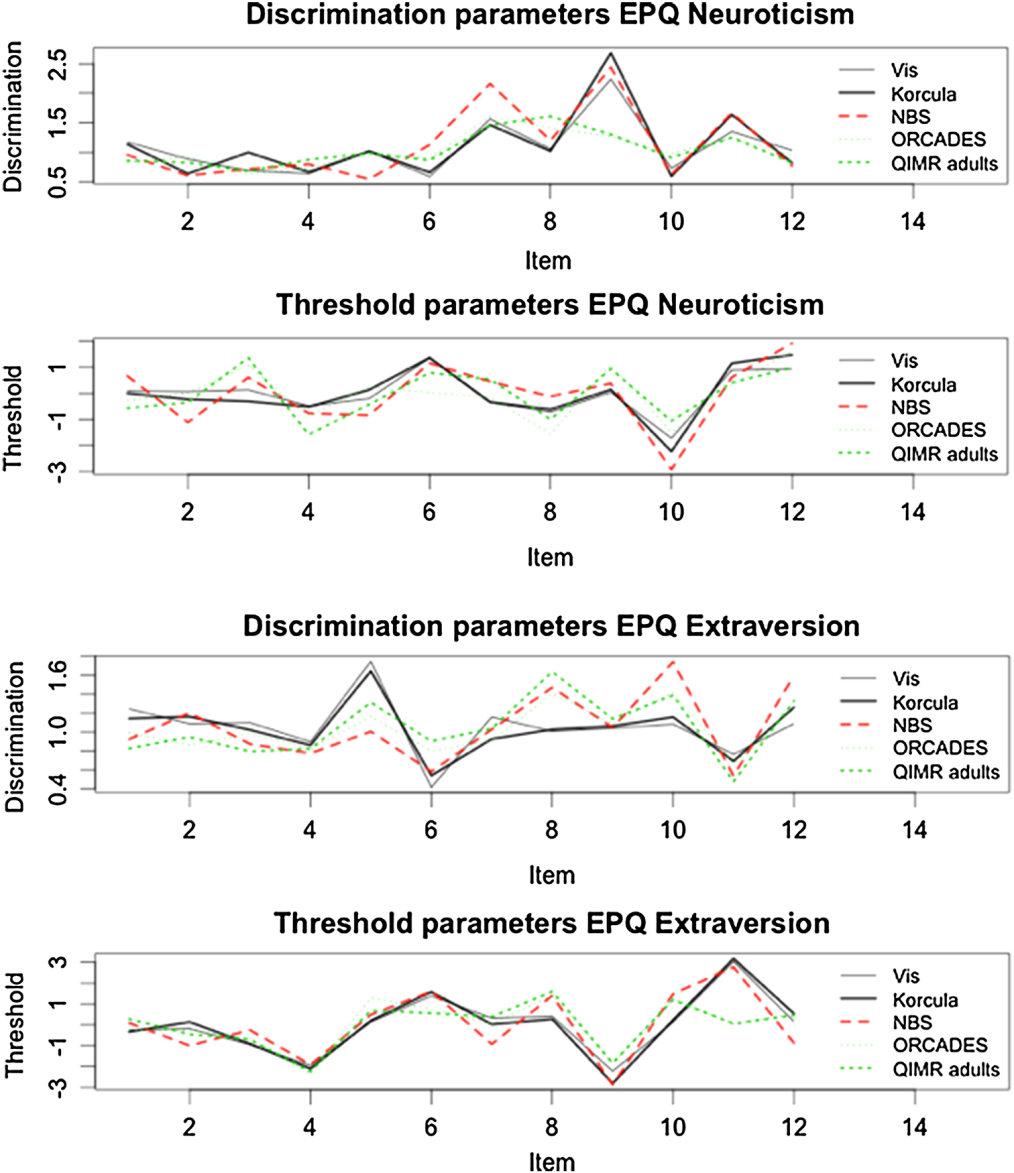
Parameter estimates (thresholds and discrimination parameters) for 12 items (*x*-axis) from the EPQ-R personality inventory for different cohorts, separately for Neuroticism and Extraversion. In *black*, the item parameter values for Croatian cohorts, in *green* for English language cohorts, and in *red* for a Dutch language cohort (Color figure online)

Figures 2 and 3 display item parameter values for the NEO-FFI and EPQ-R Neuroticism and Extraversion items for all cohorts in which these inventories were assessed. These parameters are based on a Bayesian hierarchical analysis (Verhagen and Fox 2013a, b) which takes into account any potential mean and variance differences across cohorts. All Bayes factors were smaller than 0.3. However, the item parameters were largely the same across cohorts for most items, with few striking differences. Item parameters tend to be more similar when cohorts have the same language. An example is NEO-FFI Neuroticism item 9 (‘At times I have been so ashamed I just wanted to hide’) for which the two Finnish cohorts show somewhat different item parameter values compared to the other cohorts. Examples from the NEO-FFI Extraversion scale are items 10 (‘I don’t consider myself especially “light-hearted” (R)) and 11 (‘I am a cheerful, high-spirited person’) that show differences across English speaking (red lines) and Dutch speaking cohorts (green lines). Similarly for the EPQ-R items, where item parameters for the Croatian cohorts (black lines) are very similar, as are the parameters for the English-speaking cohorts (green lines), with clear differences between the two language groups. This suggests some evidence for measurement variance across cohorts, which could be due to slightly different item content after translation.

Allowing for these significant deviations from measurement invariance across cohorts by applying the Bayesian model, Tables 3 and 4 show uncorrected means and variances per cohort, as measured by the NEO-FFI and EPQ-R items. Note that we included all cohorts with NEO data (NEO-PI-R or NEO-FFI), but using only the 12 items that are part of both the NEO-PI-R and the NEO-FFI. NESDA shows the highest mean Neuroticism score (which is expected given that it concerns a sample selected for depression and anxiety) and PAGES the lowest mean for NEO data. For NEO Extraversion, the QIMR adolescents show the highest mean (as expected based on their age), and CILENTO the lowest mean. Based on the EPQ data, the Croatian samples have the highest Neuroticism and Extraversion scores, and ORCADES the lowest. Some variance differences across cohorts are also observed, which can partly be explained by differences in age distribution, birth cohort and inclusion criteria. Note that for the NEO, the variances for Neuroticism are larger than for Extraversion, which is explained by the higher reliability of the Neuroticism scale. This is because in the hierarchical modeling, in order to identify scale, the product of the discrimination parameters was fixed at 1, both for Neuroticism and for Extraversion. Larger variance of the latent trait implies that in case the latent variance was fixed to a constant instead of the discrimination parameters, the discrimination parameters would be higher for Neuroticism than for Extraversion. As these discrimination parameters are used for computing test information (Lord 1980), and therefore reliability, we can conclude that Neuroticism is more reliably assessed than Extraversion.

**Table 3:** Estimated means and variances of IRT-based Neuroticism and Extraversion latent scores based on NEO-FFI item data, after taking into account measurement non-invariance across cohorts

	Neuroticism		Extraversion	
Cohort	Mean (SE)	Variance	Mean (SE)	Variance
2. BLSA	−0.93 (0.04)	0.93	0.50 (0.03)	0.56
3. CILENTO	−0.14 (0.03)	0.43	−0.15 (0.04)	0.25
4. COGEND	−0.45 (0.03)	0.69	0.40 (0.03)	0.39
5. ERF	−0.28 (0.02)	0.38	0.06 (0.03)	0.23
6. EGCUT	−0.16 (0.03)	0.37	0.04 (0.04)	0.11
7. FINNISH TWINS	−0.41 (0.04)	0.74	0.34 (0.03)	0.41
8. HBCS	−0.59 (0.04)	0.65	0.13 (0.06)	0.37
11. LBC1936	−0.77 (0.04)	1.10	0.25 (0.03)	0.50
14. NESDA	0.05 (0.04)	1.12	0.03 (0.03)	0.62
15. NTR	−0.69 (0.04)	0.88	0.57 (0.03)	0.55
17. PAGES	−1.02 (0.05)	0.74	0.28 (0.07)	0.50
18. QIMR adolescents	−0.11 (0.03)	0.60	0.68 (0.03)	0.49
19. QIMR adults	−0.43 (0.03)	0.81	0.36 (0.03)	0.40
23. YOUNG FINNS	−0.73 (0.04)	1.24	0.50 (0.03)	0.61
Overall average	−0.47 (0.09)	0.12^a^	0.28 (0.07)	0.07^a^

^a^Between cohort variance

**Table 4:** Estimated means and variances of IRT-based Neuroticism and Extraversion latent scores based on EPQ-R item data, after taking into account measurement non-invariance across cohorts

	Neuroticism		Extraversion	
Cohort	Mean (SE)	Variance	Mean (SE)	Variance
9. Korcula	−0.55 (0.06)	2.28	1.41 (0.07)	2.10
13. NBS	−1.33 (0.07)	2.94	0.60 (0.07)	3.52
16. ORCADES	−1.47 (0.08)	2.56	0.36 (0.08)	3.10
19. QIMR adults	−0.72 (0.06)	2.35	0.76 (0.07)	4.12
22. VIS	−0.33 (0.06)	2.22	1.10 (0.06)	2.02
Overall average	−0.83 (0.23)	0.30^a^	0.82 (0.21)	0.23^a^

^a^Between cohort variance

### Meta-analysis of heritability

MZ twin correlations for the estimated Neuroticism and Extraversion scores ranged between 0.39 and 0.54 (Table 1). DZ correlations were typically smaller than half the MZ correlations, suggesting non-additive genetic effects on variation in Neuroticism and Extraversion. Significant sex differences in same-sex twin correlations (*p* value <0.01) were found in the MCTFR, Finnish Twin and STR cohorts, but not in the NTR and two QIMR cohorts. The NTR and QIMR cohorts included opposite-sex twins. Table 1 shows that in the NTR and in the QIMR adolescent cohorts, the opposite-sex twin correlations are not significantly different from the same-sex DZ twin correlations, nor are the male same-sex DZ twin correlations different from the female same-sex DZ twin correlations. Only in the QIMR-adult cohort, there is some evidence of a larger same-sex DZ correlation for Neuroticism in females compared to males.

In the meta-analysis of the 27 twin correlations in Table 1, the base model for Neuroticism with 5 parameters (*h*_m_, *h*_f_, *d*_m_, *d*_f_, and one for allowing the opposite-sex twin correlation to differ from its expectation under the hypothesis of no qualitative sex differences) did not show a better fit than one where the opposite-sex twin correlation was equated to its expected value (total N = 29,496 pairs). The base model χ^2^ was 88.33, and the restricted model χ^2^ was 88.89, a non-significant change with 1 degree of freedom. Next, this restricted model with qualitatively the same additive and non-additive genetic effects for males and females was compared with a model that specified that the proportions additive and non-additive genetic variance were equal across sexes. This model had a χ^2^ statistic of 91.63, a non-significant increase of the χ^2^ statistic by 2.74 for 2 degrees of freedom. Next, it was tested whether the non-additive genetic effects could be dropped from the model. The χ^2^ statistic increased to 170.39, which is highly significant. Thus, for Neuroticism, both additive and non-additive genetic effects seem to be operating, which seem to be the same in males and females, and of equal importance in males and females. Proportions of additive and non-additive genetic variance were estimated at 27 and 21 %, respectively.

For Extraversion (total N = 29,501 pairs), the base model had a χ^2^ of 97.15. Restricting the opposite-sex twin correlation led to a χ^2^ of 104.67, a difference of 7.54, which is significant at one degree of freedom. We therefore allowed for qualitative sex difference when testing for quantitative sex differences (equating *h*_m_ to *h*_f_, and *d*_m_, to *d*_f_,). This restriction led to a χ^2^ of 101.20, a non-significant change of 4.06 at 2 degrees of freedom, *p* = 0.13. Thus, there seem to be only qualitative differences in genetic variance components. Dropping non-additive genetic variance from the model resulted in a significantly higher χ^2^ statistic, of 194.60, a difference of 93.40.

Thus, for Extraversion, there are qualitative sex differences in the additive and non-additive genetic effects, but the additive and non-additive genetic effects are of equal magnitude in males and females: 24 % and 25 %, respectively. The χ^2^ statistic for these qualitative sex differences was relatively small given the large sample size, but nevertheless, the opposite sex twin correlation was a factor 0.76 smaller than expected under no qualitative differences (i.e., 0.14 instead of 0.18).

### Power study

For the NTR cohort, the statistical power to detect a SNP at the genome-wide significance level that explains 0.1 % of the true phenotypic variance (latent trait) with an allele frequency of 0.5 when using only the 12 NEO-FFI items was 18 % (N = 5,299 individuals with NEO-FFI data on Neuroticism) and increased to 44 % when using IRT scores based on both NEO-FFI and ABV data (N = 31,309 individuals with either NEO-FFI data, ABV data or both). In the QIMR-adult sample, the power with only 12 NEO-FFI items was 0 % (N = 3,712). Using all available data from all inventories and analyzing IRT scores yielded a power of 30 % (N = 26,692). Thus, the power in GWAS substantially increases if item data from multiple inventories are included, if available.

## Discussion

This study examined for Neuroticism and Extraversion personality traits whether measures from different inventories could be harmonized using IRT test linking. The IRT analyses showed that the linked scores for Neuroticism and Extraversion that were estimated in >160,000 individuals from 23 cohorts were largely independent of the particular inventory. The success of this approach is demonstrated by the power study that showed a clear increase in statistical power to find a genetic variant associated with personality that is mainly the result of an increase in sample size.

The NEO, Eysenck and IPIP inventories were especially conducive to being linked. Linking was slightly less successful for TCI and MPQ with the NEO, Eysenck and IPIP inventories. The mapping of Harm Avoidance onto Neuroticism, despite theoretical differences between the concepts, was found to be relatively good. However, the mapping of Reward Dependence to Extraversion was less feasible, as was suspected. Such imperfect linking results in bias when individuals are ranked, which is very important in for example educational settings (e.g. pass/fail decisions on a test or determining the final class rank). However, when scientific interest is in population effects, like a correlation in twins or between the phenotype and a SNP, results are highly satisfactory. When dealing with non-identical but correlated traits, an alternative could be the use of multidimensional IRT models (van den Berg and Service 2012), because such models allow for relatively low correlations between multiple latent construct, but still enable borrowing statistical information from the respective sets of items, which leads to more precise estimation of latent scores.

Across cohorts, personality scores were largely comparable; that is, the extent of measurement variance was overall not large. We did, however, observe measurement variance for a few cohorts and for some items. Differences in item parameters across cohorts seem largest in cohorts with different spoken languages, suggesting cultural and/or language effects on some of the items. As a consequence, the estimated latent scores across cohorts are not based on completely identical scales. Again, for individual scoring this has consequences (e.g., a person’s ranking within a population), but these imperfections have little effect on results for population effects, because the correlation of two scores based on different calibrations was generally very high. Overall, the conclusion is that data pooling within cohorts and subsequently pooling results across cohorts in a meta-analysis is meaningful for Neuroticism and Extraversion and these inventories. As the power study showed, such pooling of data within cohorts can lead to a potentially large increase in statistical power. Such increase in power is largely due to the increase in sample size, but also of using more phenotypic information per individual.

Note that IRT test linking is always *possible*: the only requirement is that there is either overlap in individuals that were administered several inventories, or overlap in items, when some items are present in multiple inventories. It remains however to be determined whether the linking leads to psychometrically sound re-scaled phenotypes in order to for the test linking to be meaningful and successful.

Based on six cohorts with twin data, the meta-analysis broad-sense heritability was 48 % for Neuroticism and 49 % for Extraversion (total N = 29,496 and 29,501 twin pairs, respectively). There was clear evidence of non-additive genetic variance for both traits. Although this finding could be partly due to a scale effect (the test information curves are slightly skewed, so therefore the distributions of sum scores *and* IRT score estimates are skewed as well, see (van den Berg et al. 2007; van den Berg and Service 2012), the relatively large size of the dominance genetic variance component suggests there is truly non-additive gene action. Sex differences in the kind of, and the relative size of, genetic factors on Neuroticism and Extraversion were suggested in only a subset of cohorts. The meta-analysis showed that qualitative sex effects were only significant for Extraversion. Proportions of additive and non-additive genetic variance were not significantly different across sexes.

We reported high correlations among the IRT-based scores and the sum scores for the specific personality inventories. One may argue that sum scores can serve just as well in analyses. There are several reasons however why the IRT approach is superior. First, the IRT approach leads to less biased estimates for Neuroticism and Extraversion if not exactly the same set of items is administered to all individuals, as was often the case in the cohorts because of missing data or because of assessing multiple inventories or versions. In addition, the IRT approach results in increased measurement precision for individuals who have been assessed using multiple inventories. Without fitting an IRT model, it is not clear how to weigh items from different inventories. Moreover, by using IRT, groups of individuals within cohorts with different item sets can be compared since all individuals are scored on one common metric, once linking is possible. Lastly, and most importantly, the IRT approach enables one to make explicit the extent to which item data from multiple inventories can be combined, both within and across cohorts. When simply using sum scores for different inventories separately and pooling results, it remains unknown whether this is actually appropriate.

When estimating latent trait scores, we preferred linking inventories within cohorts, but not across cohorts. Arguably, linking across cohorts would be even better, scaling all individuals from all cohorts to one common metric. Although theoretically possible, it can be infeasible in practice. In our study, it would require analyzing hundreds of items in over 160,000 individuals in one analysis, which is computationally infeasible. This approach would also only be possible if all inventories could in fact be linked to one another. In our study, this was not the case; for instance, different versions with different answer categories of the same inventory were used in different cohorts.

Limitations of the current study are that we did not include all items in cases of repeated measures, item data were assumed to be missing at random (Little and Rubin 1989), and we preselected items to belong to Neuroticism or Extraversion, rather than making this choice data-driven. Future extensions of the IRT linking approach may address these issues. Also note that our method deals with harmonization of continuously distributed data. Generally, harmonization of case–control status requires a different approach, but in cases where diagnosis is based on cut-off scores on continuous measures (e.g. a symptom count), the application of IRT models could prove helpful; IRT models are also used to compare pass/fail decisions in educational measurement where students are differentially assessed.

To conclude, the IRT results show that the Neuroticism and Extraversion item data from different inventories in different cohorts can be harmonized (for general recommendations and an example R analysis script, see Supplementary Materials Online). The harmonized phenotypes can now also be confidently correlated with brain measures or used in a GWA study. The IRT analysis is not only useful for harmonizing phenotypes, it is also informative regarding the power to find significant genetic variants of various allele frequencies. The TICs show where in the distribution of Neuroticism and Extraversion scores there is most phenotypic information. Relating these TICs to the power a phenotypic test might give in a GWAS (van den Berg and Service 2012), we conclude that there generally is more power to detect low frequency genetic variants associated with scoring at the low end of the Extraversion distribution than towards the high end of the distribution. Similarly, there is more power to detect low-frequency genetic variants associated with scoring above-average on Neuroticism, compared to scoring below-average. Overall, the phenotypic information content is higher for Neuroticism than for Extraversion in most cohorts, suggesting more power to find loci for Neuroticism than for Extraversion. Combined with the finding of more additive genetic variance in Neuroticism than in Extraversion, we expect that Neuroticism loci will be easier to find than Extraversion loci.

## Electronic supplementary material

Below is the link to the electronic supplementary material.

Supplementary material 1 (DOCX 1159 kb)
