# Supplementary material for: Harmonization of Neuroticism and Extraversion phenotypes across inventories and cohorts in the Genetics of Personality Consortium: an application of Item Response Theory
Source: Behav Genet. 2014 May 15;44(4):295–313. doi: 10.1007/s10519-014-9654-x (PMC4057636; doi:10.1007/s10519-014-9654-x)
Supplement: Supplementary file 1 — Supplementary material 1 (DOCX 1159 kb) [file 10519_2014_9654_MOESM1_ESM.docx]

**Supplementary Materials Online**

Belonging to **Van den Berg, de Moor et al. *Harmonization of Neuroticism and Extraversion Phenotypes across Inventories and Cohorts in the Genetics of Personality Consortium: an Application of Item Response Theory***

**Materials and methods**

**Cohorts**

*1. ALSPAC (Boyd et al. 2013) — United Kingdom.* The Avon Longitudinal Study of Parents and their Children (ALSPAC) is a longitudinal population-based birth cohort that recruited pregnant women residing in Avon, UK, with an expected delivery date between 1st April 1991 and 31st December 1992. 14 541 pregnant women were initially enrolled with 14 062 children born. Biological samples including DNA have been collected for 10 121 of the children from this cohort. Ethical approval was obtained from the ALSPAC Law and Ethics committee and relevant local ethics committees, and all parents provided written informed consent. In this study, 6 076 children (3 099 females; 51.0%) for whom the IPIP data were available were included. Mean age of the sample was 13.8 years (SD=0.21). The data were collected between 2005 and 2006. The study website contains details of all the data that is available through a fully searchable data dictionary (http://www.bris.ac.uk/alspac/researchers/data-access/data-dictionary).

*2. BLSA (Terracciano et al. 2005) — United States of America.* The Baltimore Longitudinal Study of Aging (BLSA) is an ongoing multidisciplinary study of community-dwelling volunteers. For this study, we examined data from 1 917 participants (952 women) of European descent that completed the NEO-PI-R questionnaire. In this sample, mean age was 58.3 years (SD=16.6). The mean age of the men was 56.0 years (SD=16.7) and of the women 60.7 years (SD=16.3). The data were collected between 1991 and 2010.

*3. CILENTO (Colonna et al. 2007; Colonna et al. 2009) —Italy.* The Cilento study is a population-based study that includes 2 137 individuals from three isolated populations of South Italy. Data from the NEO-PI-R questionnaire were available for 800 participants representing the final sample. Of this sample, 64.4% were women. The mean age of all participants was 54.6 years (SD=19), of the men 54.6 years (SD=19.2) and of the women 54.6 years (SD=19.5). The data were collected between 2009 and 2011.

*4. COGEND (Bierut et al. 2007; Saccone et al. 2007) — United States of America.* COGEND was initiated in 2001 as a three-part program project grant funded through the National Cancer Institute (NCI; PI: Laura Bierut). The three projects included a study of the familial transmission of nicotine dependence, a genetic study of nicotine dependence, and a study of the relationship of nicotine dependence with nicotine metabolism. The primary goal is to detect, localize, and characterize genes that predispose or protect an individual with respect to heavy tobacco consumption, nicotine dependence, and related phenotypes and to integrate these findings with the family transmission and nicotine metabolism findings. As a part of this study, the NEO-FFI was administered to 2 712 participants (1 679 women; 61.9%) and all participants completed the test. In this sample, mean age was 36.6 years (SD=5.6). The mean age of the men was 36.3 years (SD=5.7) and of the women 36.7 years (SD=5.5). The data were collected between 2003 and 2007.

*5. EGCUT (Metspalu 2004) — Estonia*. The Estonian cohort comes from the population-based biobank of the Estonian Genome Project of University of Tartu (EGCUT). The project is conducted according to the Estonian Gene Research Act and all participants have signed the broad informed consent (www.biobank.ee). In total, 52 000 individuals aged 18 years or older participated in this cohort (33% men, 67% women). General practitioners (GP) and physicians in the hospitals randomly recruited the participants. A Computer-Assisted Personal interview was conducted during 1–2 h at doctors’ offices. Data on demographics, genealogy, educational and occupational history, lifestyle and anthropometric and physiological data were assessed. The personality profile was assayed using NEO-PI-3 questionnaire and was administered to 1 730 participants. In this sample, the age range was 18–88 years (M=42.8 years, SD=16.5). The sample consisted of 740 men (mean age 42 years, SD=16.3) and 991 women (mean age 43.4 years, SD=16.6). The data were collected between 2009 and 2012.

*6. ERF (Pardo et al. 2005) — The Netherlands.* The Erasmus Rucphen Family (ERF) study is a family-based study including over 3 000 individuals from an isolated population in the Southwest region of the Netherlands. There were 2 400 individuals for whom both NEO personality and GWA data were available. The mean age of all participants was 49.3 years (SD=14.9) and women constituted 55.8% of the total sample (M=49.0, SD=15.1, versus in men M=49.6, SD=14.7).

*7. FINNISH TWINS (Kaprio 2006; Kaprio 2013) — Finland.* The Finnish twin cohort consisted of 30 654 respondents, of which 28 767 completed the Eysenck Personality Inventory (EPI; an alternative version of the EPQ) at least once. EPI was assessed in 1975-1976 and for the second time in 1981-1983). The NEO-FFI was assessed between 2003-2009 for the index twins of the Nicotine Addiction Genetics - Finland study and wave 4 of the FinnTwin12 study. Because of the large time difference between EPI and NEO-FFI assessments (20-33 years), EPI and NEO-FFI item data were analyzed separately to estimate latent personality scores. The age range of this sample at the time of EPI assessments was 18-95 years (mean age 36.4, SD=14.6), and 50.5% were women. The age range of this sample at the time of NEO assessments was 20-76 years (mean age 38.0, SD=16.7), and 46.2% were women.

*8. HBCS (Barker et al. 2005; Eriksson et al. 2006; Raikkonen et al. 2008) — Finland.* The Helsinki Birth Cohort Study (HBCS) is composed of 8 760 individuals born between the years 1934 and 1944 in one of the two main maternity hospitals in Helsinki, Finland. Between 2001 and 2003, a randomly selected sample of 928 men and 1 075 women participated in a clinical follow-up study with a focus on cardiovascular, metabolic and reproductive health, cognitive function and depressive symptoms. In 2004, various psychological phenotypes were assessed, including the NEO and TCI personality dimensions. There were 1 698 participants that completed either the NEO and/or the TCI (55.9% women). The mean age of the subjects was 63.4 years (SD=2.9). The mean age of the men was 63.3 years (SD=2.7) and of the women was 63.5 years (SD=3.0).

*9. KORCULA (Polasek et al. 2009) — Croatia.* This study was performed in the eastern part of the island of Korčula, Croatia between March and December 2007. Healthy volunteers aged 18 and over from the town of Korčula and villages Lumbarda, Žrnovo, and Račišće were invited to the study. There was a total of 969 participants included who had a number of quantitative phenotypic traits measured. The EPQ-R was successfully administered to 810 participants (511 female; 63.1%). The mean age was 55.4 years (SD=13.3; female M=54.5, SD=12.8, male M=56.9, SD=14). The data were collected in 2007.

*10. LBC1921 (Deary et al. 2011) — United Kingdom.* The Lothian Birth Cohort 1921 (LBC1921) study consists of a cohort of 550 individuals born in 1921. Most participants lived independently in the Lothian region (Edinburgh city and surrounding area) of Scotland. The majority of participants took part in the Scottish Mental Survey of 1932. Of 498 participants who were approached from the 550 original participants, there were 478 participants (283 women; 59.2%) who successfully filled in the IPIP. The mean age of these participants was 81.2 (SD=.3) and was the same for both sexes. The IPIP was administered twice: data were collected in 2002 and between 2007 and 2008. The first IPIP assessment was used for 472 individuals, and the second assessment was used for six individuals.

*11. LBC1936 (Deary et al. 2011; Deary et al. 2007; Deary et al. 2004) — United Kingdom.* The Lothian Birth Cohort 1936 (LBC1936) study consists of a cohort of 1 091 individuals born in 1936. Most participants lived independently in the Lothian region (Edinburgh city and surrounding area) of Scotland. The majority of participants took part in the Scottish Mental Survey of 1947. There were 963 participants (489 women; 50.8%) who completed the NEO-FFI and the IPIP. The mean age of these participants was 69.6 years (SD=.83; men and women equal) at the time when the IPIP and NEO-FFI was administered, and 72.5 (SD=.71; women M=72.5, SD=.70, men M=72.5, SD=.72) at the time when the IPIP was re-administered. The IPIP data were collected between 2004 and 2007, and between 2007 and 2010. From the first wave 963 administrations were used, and from the second wave 69 administrations.

*12. MCTFR (Iacono and McGue 2002; McGue et al. 2007) — United States of America.* Data from the Minnesota Center for Twin and Family Research (MCTFR) were collected as part of two different longitudinal studies, the Minnesota Twin Family Study (MTFS) and the Sibling Interaction and Behavior Study (SIBS). The MTFS is a study of reared-together, same-sex twins and their parents, and the SIBS is a study of families of different types (some include adopted offspring). Both parents and offspring completed the Multidimensional Personality Questionnaire (MPQ) at baseline, and only offspring completed it at subsequent follow-ups of approximately 3-year intervals. There were data available for up to 5 follow-ups for offspring in the MTFS and up to 3 for offspring in the SIBS. The total sample with MPQ data included 9 071 participants (53% female). The data were collected between 1998-2004, 2003-2008, and 2006-2010. The mean age of this combined sample was 33.4 years (SD=15.1). The mean age of men was 32.4 years (SD=14.7) and of the women 34.5 years (SD=15.5). Contrary to the other studies with repeated measure data of personality, we first selected the least recent item data. This strategy was thought optimal because at baseline the number of subjects for MCTFR was considerably larger than at later time points.

*13. NBS (Kiemeney et al. 2008) — The Netherlands.* In 2000 a study was initiated among the inhabitants of the municipality of Nijmegen by different departments of the Radboud University Nijmegen Medical Centre to research the question what the prevalence of certain risk factors, chronic diseases and genetic variations in the general population are. As a part of this study, the EPQ-R was administered to 1 832 participants. From this sample, 1 823 participants (921 female; 50.5%) completed the test. The mean age of these participants was 61.5 (SD=10.3; women M=56.7, SD=10.8, men M=66.3, SD=7.0).

*14. NESDA (Penninx et al. 2008) — The Netherlands.* The NESDA data for the present study were drawn from the Netherlands Study of Depression and Anxiety(Penninx et al.), an ongoing longitudinal cohort study aimed at examining the long-term course of depressive and anxiety disorders in different health care settings and phases of illness. A total of 2 981 respondents were recruited from primary care (n=1 610), specialized mental health care (n=807) and the community (n=564), including healthy controls, respondents with subthreshold symptoms and those with an anxiety and/or depressive disorder. The NEO-FFI was successfully administered to 2 961 participants (1 979 female; 66.8%). The mean age was 41.9 years (SD=13.1; female M=41.1, SD=13.1, male M=43.4, SD=12.9). Baseline data were collected between 2004 and 2009. The NEO-FFI was administered twice, at baseline and two years later. For the NESDA sample, contrary to the other studies with repeated measure data of personality, we first selected the least recent item data. For NESDA, this strategy was deemed most suitable because the first measurement represented the baseline measurement for NESDA after which treatment of cases may have followed.

*15. NTR (Boomsma et al. 2006; Boomsma et al. 2002) — The Netherlands*.

Data on personality in the Netherlands Twin Register (NTR) were collected as part of a longitudinal study on health, personality and lifestyle in adolescent and adult twins and their relatives (i.e., their non-twin siblings, parents, spouses and children). Eight waves of data collection have been completed (in 1991, 1993, 1995, 1997, 2000, 2002, 2004 and 2009). Twins were invited to participate at all time points, while the parents and siblings could participate on a maximum of 6 time points, spouses on 4 time points and adult children of twins and siblings on 2 time points. The ABV was administered five times, in 1991, 1993, 1997, 2000 and 2002, and the NEO-FFI was assessed twice, in 2004 and 2009. Of the 31 694 individuals who participated at least once in one of these seven waves, there were 31 259 individuals (58.7% female) with valid personality data (at least one Neuroticism or Extraversion item was available on at least one time point). For the analysis in this study, we selected for each individual the ABV item data of the latest time point and the NEO item data of the earliest time point. This ensured that for each individual with data on both the ABV and NEO, the times of measurement were as close as possible. For 21 146 individuals there were NEO data available (of which from 14 880 individuals data came from the 2004 survey and from 6 266 individuals data came from the 2009 survey). For the ABV, data of 6 778 individuals came from survey 2002, 1 803 from 2000, 5 088 from the 1997, 2 208 from 1993, and 2 939 from 1991 (in total 18 816 individuals with ABV data). The mean age of the participants was 37.2 years (SD=15.3) across assessments.

*16. ORCADES (McQuillan et al. 2008) — United Kingdom.* The Orkney Complex Disease Study (ORCADES) is a genetic epidemiology study based in an isolated population in the north of Scotland. It aims to discover the genes and variants in them that influence the risk of common, complex diseases such as diabetes, osteoporosis, stroke, heart disease, myopia, glaucoma, chronic kidney and lung disease. As a part of this study, the EPQ-R was administered to 602 participants (347 female) and all participants all completed the test. The mean age of these participants was 56.8 (SD=13.8; women M=56.5, SD=13.9, men M=57, SD=13.8). The data were collected between 2007 and 2011.

*17. PAGES (van den Oord et al. 2008) — Germany.* In this German cohort, healthy control participants were randomly selected from the general population of Munich, Germany, and contacted by mail. Several screenings were conducted before the volunteers were enrolled in the study. These included screening of medical and psychiatric disorders (in particular psychotic disorders) in the participants and their first-degree relatives by phone and interview and screening for central nervous system and cognitive impairment by neurological examination and cognitive testing. Furthermore, only participants with German descent (all four grandparents German) could participate. In the resulting sample, a large battery of personality questionnaires was administered as well as data on life events and traumatic events. Data on the NEO-PI-R and TCI were analyzed for the current study. There were 476 individuals (55.7% women) with valid personality data. The mean age of the sample was 45.9 years (SD=15.4; women M=43.4, SD=15.3, men M=49, SD=15.3). The data were collected between 1998 and 2006.

*18. QIMR adolescents — Australia* Data from Australian adolescents were collected in twin family studies conducted at the QIMR Berghofer Medical Research Institute (QIMR). Participants were mainly recruited through primary and secondary schools in Queensland for studies of melanocytic naevi (moles)(Aitken et al. 1994). JEPQ and/or NEO personality data (NEO-PI-R or NEO-FFI) were collected as part of the melanocytic naevi study (1992-ongoing), the cognition study (in-person testing, 1996-2012)(Wright and Martin 2004), a health and well-being study (a mail/phone study 2002-2003)(Wright and Martin 2004), and a study of borderline personality disorder (online/paper survey 2003–2006)(Distel et al. 2008). JEPQ data were available at 3 time points, NEO-PI-R data at 1 time point, and NEO-FFI data at 2 time points. We first selected the NEO and JEPQ data from the earlier time points, and subsequently selected the data from more recent time points. Personality data were available for 4,100 individuals (51.5% female). Participants ranged in age from 9 to 29 years (M=14.4, SD=2.4). The data were collected between 1992 and 2011.

*19. QIMR adults — Australia* Data from Australian adults were collected in twin family studies conducted at the QIMR Berghofer Medical Research Institute. NEO personality data (NEO-PI-R or NEO-FFI) were collected from a series of studies conducted collaboratively by Nick Martin and Andrew Heath between 2001 and 2006 (Pergadia et al., 2009; Saccone et al., 2007; Distel et al., 2008). The EPQ data were obtained from the following sources: (a) The Canberra study (1980-1981) (Heath et al., 1988): twins drawn from the Australian Twin Registry and born prior to 1964 (‘Cohort 1’); (b) Two twin studies (1988-1991) in which Health and Lifestyle Questionnaires were sent to the members of Cohort 1 and an additional group born from 1964 to 1971 (‘Cohort 2’); with similar questionnaires also sent to immediate family members of the twins (Hansell et al., 2008); (c) The Anxiety and Depression study (assessed twice, once by questionnaire and once by telephone interview) (Kirk et al. 2000) drawn from Cohort 1 and Cohort 2 but selected to include mainly individuals with extreme high or low neuroticism scores from the studies in (b) and members of their immediate families. The TCI data were obtained from two twin studies (1988-1991) from Cohort 1 and 2, and the MPQ data as part of the Gambling Study (cohort 2) (Slutske et al. 2009). Altogether, the EPQ was administered four times, the NEO-FFI twice, and the TCI, NEO-PI-R and MPQ once. We first selected the item data of the EPQ at the first assessment, because the EPQ data were available for the majority of the subjects, the TCI data was obtained at the same time point and the MPQ assessment was close to the EPQ and TCI time points. Subsequently, we selected those time points with NEO item data that were closest in time to the EPQ and TCI assessment. Data collections were approved by the QIMR Human Research Ethics Committee and informed consent was obtained from all participants. Personality data were available for 26,698 individuals (57.1% female). Participants ranged in age from 16 to 96 years (M=40.1, SD=15.0). The data were collected between 1980 and 2007.

*20. SAGE-COGA (Foroud et al. 2000; Reich et al. 1998) — United States of America*. The Study of Addiction: Genetics and Environment (SAGE) is part of the Gene Environment Association Studies initiative funded by the National Human Genome Research Institute. The sample used in this study consisted of 649 participants drawn from the Collaborative Study on the Genetics of Alcoholism (COGA) that all completed the TCI. COGA is a multi-site study funded by the National Institute on Alcohol Abuse and Alcoholism and National Institute on Drug Abuse that aims to characterize the familial transmission of alcoholism and related phenotypes and identify susceptibility genes. The mean age of all participants was 40.8 years (SD=10.79) and women constituted 45.6% of the total sample (M=40.9, SD=10.4, versus in men M=40.8, SD=11.1). The data were collected between 1991 and 1998.

*21. STR (Floderus-Myrhed et al. 1980) — Sweden.* For the Swedish Twin Registry (STR), in 1970 a cohort of twins born in 1926–67 was compiled, by use of nationalized birth registrations. A birth register consisting of all 50 000 twin births was established. Members of like-sexed pairs from the cohort born in 1926–58 were sent out a questionnaire in 1972–73. Responses were received from 36 535 individuals including 14 000 twin pairs. The EPI was included to assess personality and completed by 30 276 individuals (52.3% female). Information is maintained concerning both the initial birth cohort as well as the subsample of like-sexed pairs from which the questionnaire information was obtained. Participants in this cohort ranged from 13 to 46 years of age when the test was administered. The mean age was 28.7 years (SD=9.1). The mean age of the men (N=14 462) was 28.4 years (SD=9.1) and of the women (N=15 839) 28.9 years (SD=9.1). The data were collected in 1972.

*22. VIS (Ivkovic et al. 2007) — Croatia.* Adult participants living in the villages of Komiza and Vis on the Croatian island of Vis were recruited in May 2003 and May 2004 for a large genetic study. Croatia has 15 Adriatic Sea islands with populations greater than 1 000. The villages on the islands have unique population histories and have preserved their isolation from other villages and the outside world through many centuries. Informed consents, procedures and questionnaires were reviewed and approved by relevant ethics committees in Scotland and Croatia. All individuals over 18 years old and resident on the Island of Vis were invited to participate in this study. As a part of the interview participants also completed the Eysenck Personality Questionnaire-Revised (short-form; EPQ-R). Seventy percent of the villages’ adult population took part in the study, a total of 918 individuals (531 female; 57.8%), 9 of whom have all missing data. The mean age was 56.4 years (SD=15.5; female M=56.7, SD=16, male M=55.9, SD=14.9). The data were collected between 2003 and 2004.

*23. YOUNG FINNS (Raitakari et al. 2008) — Finland.* The Young Finns Study is an ongoing multicenter follow-up study of Finnish children and adolescents started in 1980 with a baseline sample of 3 596 individuals. Personality data were collected in 2007 from 2 058 participants of whom 2 057 were included in the study with NEO-FFI-data (one participant had all missing data), 1 212 were female (58.9%). The mean age of all participants was 37.6 years (SD=5), men and women equal (including SD).

For a more schematic overview, see **Supplementary Table 1**.

**Personality assessment**

*NEO personality inventories*

The NEO personality inventories have been developed mainly in the factor-analytic tradition.(Costa and McCrae 1992) Five higher-order traits are distinguished in the NEO inventories, labeled Neuroticism, Extraversion, Openness to Experience, Agreeableness and Conscientiousness.(Costa and McCrae 1992) Neuroticism is also known as emotional instability. It involves the experience of negative emotions such as anxiety, depression, hostility, and the vulnerability to stress. Extraversion is characterized by positive emotions, gregariousness, and the tendency to be active, seek out stimulation and enjoy the company of others. Openness to Experience involves active imagination, aesthetic attentiveness, variety preference and intellectual curiosity. Agreeableness can be defined as the tendency to be cooperative and compassionate rather than suspicious and antagonistic towards others. Lastly, the dimension of Conscientiousness reflects self-discipline, carefulness, thoroughness, organization, deliberation and achievement.

Across studies, four different versions of the NEO personality inventory were used: the revised NEO personality inventory (NEO-PI-R)(Costa and McCrae 1992), a more readable adaptation of the revised NEO personality inventory (NEO-PI-3)(McCrae et al. 2005), the NEO Five-Factor Inventory (NEO-FFI) (Costa and McCrae 1992)and the revised NEO Five-Factor Inventory (NEO-FFI-R)(McCrae and Costa 2004). The NEO-PI-R consists of 240 items measuring 30 facets (8 items per facet). The 30 facets cluster into the 5 higher order factors (also called domains). The NEO-FFI is a shortened version of the NEO-PI-R and contains a selection of 60 of the 240 items. The 60 items measure the 5 higher order factors (12 items per factor). Most studies either included the NEO-PI-R or the NEO-FFI (see **Supplementary Figure 3** and **Supplementary Table 1**). The NEO-PI-3 was assessed in the EGCUT sample (Allik et al. 2004; Kallasmaa et al. 2000) and the QIMR adolescents sample included the NEO-FFI-R.(McCrae and Costa 2004)

This study focuses on the items that measure Neuroticism and Extraversion. Thus, 48 Neuroticism and 48 Extraversion items were analyzed if the long forms of the NEO inventories were assessed, and 12 Neuroticism and 12 Extraversion items were analyzed if the short forms were assessed, with the following exceptions. In the Finnish Twins sample, the NEO-PI-R items were analyzed separately from the EPI data, that is, tests were not linked, because there was a large time lag in between assessments (20-33 years). For CILENTO, one Neuroticism and one Extraversion item were excluded because of unexplainable low correlations with the other items of the same factor. For HBCS, a subset of NEO-PI-R items was assessed and analyzed (36 for Neuroticism and 30 for Extraversion). Regardless of the specific NEO version, all items were answered on a 5-point Likert scale, with the categories 0 = “Strongly disagree”, 1= “Disagree”, 2 = “Neither agree nor disagree”, 3 = “Agree”, and 4 = “Strongly agree”. Negatively keyed items were reverse scored prior to analysis.

*Eysenck personality inventories*

Eysenck developed his inventories as part of a neurobiological theory of personality. In his theory, Eysenck at first distinguished between 2 main dimensions of personality: Neuroticism and Extraversion.(Eysenck and Eysenck 1964) Later, he revised his theory and added Psychoticism as the third factor.(Eysenck and Eysenck 1975) Definitions of Neuroticism and Extraversion in Eysenck’s theory resemble those in Big Five theories, although there are some differences. According to Eysenck’s theory, Neuroticism is associated with the limbic system: higher Neuroticism is associated with higher sensitivity to emotional stimulation. Extraversion is related to the optimal level of arousal: extraverts are under-aroused and therefore seek more stimulation, while introverts are over-aroused and tend to avoid stimulation. Psychoticism encompasses a combination of impulsivity, non-conformity, anger and aggression and sensation seeking.

Across studies, 4 different versions of Eysenck personality inventories were used: the revised Eysenck Personality Questionnaire short form (EPQ-R-S)(Eysenck and Eysenck 1975; Eysenck et al. 1985), the Junior Eysenck Personality Questionnaire (JEPQ)(Eysenck 1972), the Eysenck Personality Inventory (EPI) (Eysenck and Eysenck 1964)or the EPI-based Amsterdamse Biografische Vragenlijst (ABV)(Wilde 1970). Historically, the EPI (and thus ABV) is the oldest inventory. It measures Neuroticism, Extraversion and Lie (a measure of social desirability). Besides Neuroticism, Extraversion and Lie, the EPQ-R-S and JEPQ also measure Psychoticism. The EPQ-R-S consists of 48 items (12 items per factor). The JEPQ contains 81 items in total, of which 20 items measure Neuroticism, 24 Extraversion, 17 Psychoticism and 20 Lie. The EPI contains 10 items to assess Neuroticism and 9 items to assess Extraversion. The ABV includes 30 items that measure Neuroticism and 21 items that measure Extraversion.

For this study only the Neuroticism and Extraversion items were analyzed. Items of the Eysenck’s personality inventories could be answered with 0 = “No”, 1 = “?”, and 2 = “Yes”. Answer category 1 = “?” was recoded as missing, and 2 = “Yes” recoded to 1 in all studies. Negatively keyed items were reverse scored prior to analysis.

*Cloninger personality inventories*

Temperament and Character Inventory (TCI)(Cloninger C 1993) version 9 was used in all studies that assessed the temperaments Harm Avoidance, Novelty Seeking, Reward Dependence and Persistence. In addition to these four temperaments, the TCI also measures the three characters Self-Directedness, Cooperativeness and Self-Transcendence. The TCI consists of 240 items, of which 40 items measure Novelty Seeking, 35 Harm Avoidance, 24 Reward Dependence, 8 Persistence, and the remaining items measure the three characters or are filler items. Items could be answered in a True-False format (0 = “False” and 1= “True”). Again, we reversed the scores of negatively keyed items.

*The International Personality Item Pool Big-Five 50-item inventory (IPIP)*

The International Personality Item Pool Big-Five 50-item inventory (IPIP) is a subset of 50 items from the International Personality Item Pool aimed at measuring the Big Five personality traits Neuroticism, Extraversion, Intellect, Agreeableness and Conscientiousness (see also description of NEO personality inventories).(Goldberg 1999) Each of the Big-Five personality factors consists of 10 items. For this study, we analyzed the 10 items of Neuroticism and the 10 items of Extraversion. The IPIP has five answer categories: 0 = "Very inaccurate", 1 = "Moderately inaccurate", 2 = "Neither inaccurate nor accurate", 3 = "Moderately accurate", and 4 = "Very accurate". Negatively keyed items were reverse scored prior to analysis.

*The Multidimensional personality questionnaire (MPQ)*

The Multidimensional personality questionnaire (MPQ)(Tellegen 2000; Tellegen and Waller 2008) is a broader personality inventory that is derived from factor analysis. It measures 11 primary personality traits, which can be clustered into four higher-order factors. The higher-order factors are Negative Emotionality (NEM), Positive Emotionality (PEM), Constraint (CON) and Absorption (ABS). NEM refers to the proneness to experience negative emotions, such as anxiety, depression, anger and aggressiveness. PEM encompasses the tendency to experience feelings of joy, to be active and to be inclined to engage in rewarding social and work environments. CON includes lack of impulsivity and sensation seeking behaviors. ABS represents openness to a wide variety of absorbing and self-involving sensory and imaginative experiences. NEM corresponds most closely to Neuroticism, although NEM is s broader concept because it also includes items about aggression. PEM corresponds to Extraversion. Therefore, we decided to analyze all PEM items to obtain Extraversion scores, and all NEM items but excluding the aggression items to obtain Neuroticism scores.

Two studies assessed the MPQ: the MCTFR and the QIMR adult studies. The MCTFR included the 198-item version of the MPQ. In this version, NEM consists of 54 items (of which 18 aggression items were excluded, leaving 36 items in the analysis for Neuroticism), and PEM consists of 72 items. Answer categories were 1 = “definitely true” (or “definitely A” for some items in which respondents need to choose between two statements or alternatives), 2 = “probably true” (or “probably A”), 3 = “probably false” (or “probably B”) and 4 = “definitely false” (or “definitely B”). The QIMR adults sample included an Australian version of the 198-item MPQ. Because the content of some items was not the same as the version used in the MCTFR sample, we could only include 27 NEM items for Neuroticism and 52 PEM items for extraversion in the QIMR adult sample. Answer categories were 0 = “false” and 1 = “true”. Negatively keyed items were reverse scored prior to analysis.

**Statistical analyses**

*Item-Response Theory (IRT) models*

Item-Response Theory (IRT) models come in many shapes (Lord 1980). We will discuss the 2-parameter logistic (2PL) IRT model for dichotomous data and one of its possible extensions for polytomous data, the Generalized Partial Credit model (Muraki 1992).

Suppose we have (0,1; no/yes) data on *N* persons on *K* items. Then the probability of a person *i* answering an item *j* with ‘1’, ‘correct’ or ‘yes’ can be regarded to be dependent on a characteristic of that person *i*, theta, and to be dependent on characteristics of the item. For example, if the item is part of an IQ test, we could say that the probability of a correct answer depends both on the intelligence of the person but also on the difficulty of the item. In a two-parameter model, we not only model item difficulty, but also the extent to which an item discriminates between people of low and high intelligence. In the 2PL, the probability of a correct answer (‘1’) is modeled as logistic function of a person parameter theta, and item parameters *a* and *b*, as follows

$P\left( Y_{ij}=1 \right)=\frac{e^{a_{j}{(\theta}_{i}-b_{j})}}{1+e^{a_{j}{(\theta}_{i}-b_{j})}}$ .

Different items have different parameters *a* (discrimination) and *b* (difficulty). **Supplementary Figure 1** shows an example of item characteristic curves that show how the probability of a positive response is a function of latent trait theta for two different items 1 and 2, where item 1 has a low difficulty level (on the negative side of the scale) and relatively low discrimination, and where item 2 has a high difficulty level (on the right hand side) and very high discrimination (i.e., a steep slope). If we assume theta as standard normally distributed this would mean for an intelligence test that item 1 would be relatively easy: even people with below average intelligence (theta<0) have a reasonable probability of knowing the correct answer. Item 2 would be relatively difficult, people with below average intelligence have a near zero probability of coming up with the right answer. Item 2 also has high discrimination: nearly all people with theta<1 will have probability close to 0 and nearly all people with theta>1 will have probability close to 1 to know the answer. This discrimination is indicated by the steepness of the slope of the item characteristic curve for item 2; a small difference in latent trait value around 1 goes together with a large difference in the probability. The discrimination (parameter *a*) is therefore the IRT analog of the factor loading in the common factor model. The difficulty parameter is then interpreted as the intercept, the point on the scale of theta where the log odds of a correct answer equals zero, which is identical to the point on the scale where the probability of correct response is 50%. Since the term ‘difficulty’ is inappropriate when items have no logically correct answer, such as in the domain of personality, we can speak of *b* as the threshold parameter. Alternatively, parameter *b* can be described as indicating the ‘traitness’ of an item: some items for example may describe behavior associated with high levels of Neuroticism (leading to high thresholds or high *b* parameter values), and other items may describe neurotic-like behavior that is also shown by individuals with lower levels of Neuroticism (resulting in lower threshold or *b* parameter values). Discrimination is then the extent to which an item discriminates between individuals that score high and low on Neuroticism.

There are several extensions of the 2PL for polytomous data (say, data with response categories ‘1’, ‘2’, ‘3’, ‘4’, and ‘5’, as for NEO and IPIP data), one of which being the Generalized Partial Credit Model.(Muraki 1992) For *M* response categories, this model has *M*-1 threshold parameters and 1 discrimination parameter per item. With *M*=2, the model reduces to the 2PL. In **Supplementary Figure 2**, we see the *category* characteristic curves for Item 2 from the NEO-PI-R as calibrated in the CILENTO cohort. It shows that the probability of scoring in category 1 decreases as Neuroticism increases. At high levels of Neuroticism, category 4 is the favorite category, and only at very high levels, people start scoring in category 5. For this item, discrimination parameter *a* was estimated at 0.76 and the threshold parameters at -3.05, 0.86, -1.51 and 3.28. As can be seen in **Supplementary Figure 2**, these thresholds are the points on the scale where the probability of scoring in category *m* becomes larger than the probability of scoring in category *m*-1.


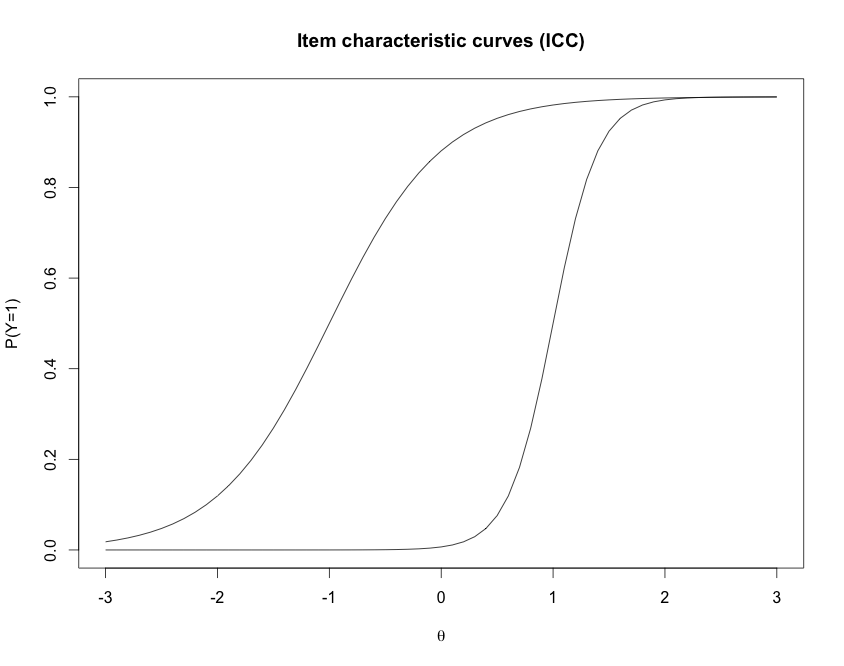


**Supplementary Figure 1**. Item characteristic curves for two items under the 2PL model. Left curve is for an item that has relatively low difficulty/low threshold (*b*=-1) and relatively low discrimination (*a*=2). Right curve is for an item that has relatively high difficulty/high threshold (*b*=1) and very high discrimination (*a*=5).


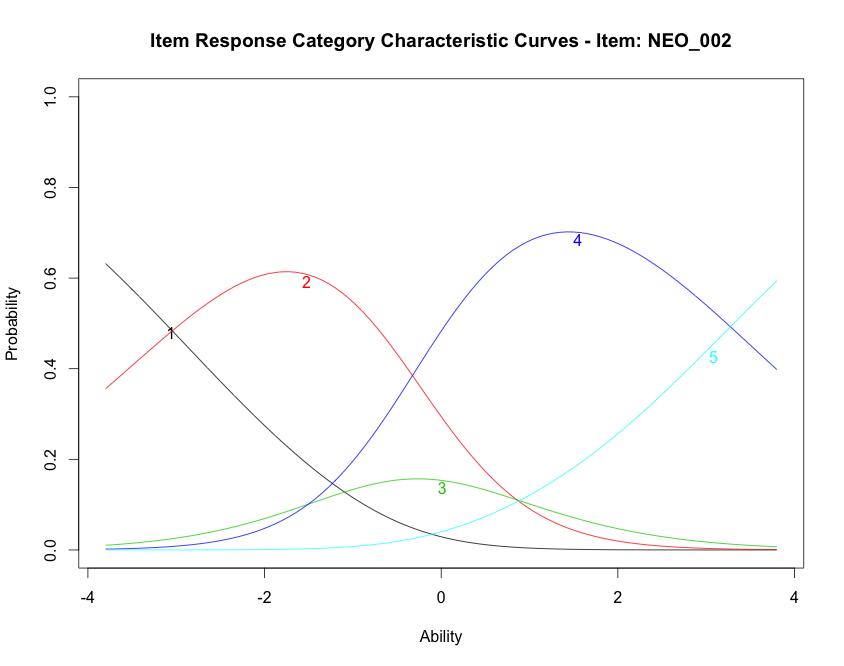


**Supplementary Figure 2**. Category characteristic curves for one of the NEO neuroticism items in the CILENTO study.

*A concrete example of the IRT logic when linking data from different tests*

Suppose Person A did test I with dichotomous items 1, 2 and 3, and Person B did test II with dichotomous items 4, 5, and 6. Because the items are very different, it is not possible to compare persons A and B solely based on their item scores. But suppose we have an additional data set on 1 000 persons that filled out both tests I and II, so that we have for every individual data on all six items. Let us assume that both tests I and II measure the same trait, that is, that the latent factor underlying items 1 through 3 correlates perfectly with the latent factor underlying items 4 through 6. Then we can assume a unidimensional IRT model for the 6 items that explains correlations among all item scores. Using the data on the 1 000 individuals with complete data, we estimate item parameters for the six items. This is called ‘concurrent calibration’, where calibration means determining a set of item parameters. **Supplementary Table 3** presents the difficulty parameters *b* and the discrimination parameters *a*. Given these calibrated item parameters, the latent trait scores for persons A and B can be estimated. These are presented in the last column with their standard errors. Estimates are also displayed for a number of persons in the complete data set, to illustrate that their standard errors are smaller since they are based on more information. As can be seen from the results, items in this example differ mainly in their difficulty parameter b: overall, Test II seems slightly more ‘difficult’ (i.e. you have to be generally more neurotic to say yes to items 5 and 6 than to say yes to items 2 and 3). Both A and B have a sum score of 2, but IRT estimates show that given the higher difficulty of test II, person B’s estimate for neuroticism is higher than person A’s estimate. Note also that person C, who was assessed using all six items and had the same item scores as A and B combined, gets a different estimate, but with a smaller standard error, since the estimate is based on more information. Further, note also that persons C and E are assessed with the same items and the same sum score of 4, but have different estimates. This is because C scores 1 on items that generally have higher discrimination values than those of the items that E scores 1 on: these items are weighted more than the items with lower discrimination values when estimating latent trait values.

*Data linking within studies*

Because some instruments had response formats with more than two answer categories (e.g., NEO and IPIP inventories), the Generalized Partial Credit Model (GPCM; Muraki 1992) was used for estimating item parameters. This was implemented in the ltm package (Rizopoulos 2006) in the statistical software program R using the gpcm() function. The same model was used for data sets with only dichotomous items in order to avoid any effects due to estimation method. Person scores were the expected a posteriori estimates (EAP)(Bock and Mislevy 1982), conditional on the observed item data for a particular person and the relevant calibrated item parameters. Prior to score estimation however, it was checked that all discrimination parameters were positive. Negative discrimination parameters are an indication that an item has not been properly (reverse-)coded. In the QIMR adult and adolescent cohorts, NEO-PI-R items 73 and 78 were omitted from the Extraversion scale since their negative parameter values could not be traced back to reverse-coding problems.

In studies where only one test was used with one measurement in time (CILENTO, Young Finns, VIS, KORCULA, EGCUT, BLSA, NBS, NTR, ORCADES, COGEND, SAGE-COGA, and ALSPAC), the IRT model was calibrated (i.e., item parameters were estimated) using the data from individuals that had no missing data. Next, conditional on the calibrated item parameters (i.e., assuming they are known), person scores were estimated using all available data. In this way, persons with some missing data received a score on the personality trait. Note that in this approach, the missing item data are assumed missing at random (Little and Rubin 1989).

In cases of multiple measurements of only one test (NESDA, LBC1921, MCTFR, ERF), data were used from the first wave, and if no item data were available for a person, data were used from the second wave, and when still not available, the third wave. Again, the IRT model was calibrated using persons with complete data, after which scores were estimated for all persons.

In the case of multiple inventories in a sample, IRT models were fit for each inventory separately, after which the model was calibrated for all inventories combined. It was checked whether item parameters did not change too much once items from other inventories were included.

There was one exception to this linking of multiple tests: in the Finnish Twins sample, the NEO-FFI items were not analyzed together with the EPI items, because of a very large time lag in between assessments (20-33 years). It is unlikely that personality is stable across such a long time period and indeed sum score correlations for NEO and EPI were very low. For the Finnish Twins, personality scores were primarily based on EPI item data, with a preference for data from the first wave. In case there were no EPI item data available, any available NEO item data were used.

In case of multiple inventories and multiple measurements for the same inventory (NTR, QIMR, LBC1936), data were used from measurement waves that were as close in time as possible to the waves of the other tests (preferably the same wave). If for a person there was no data from that particular wave, data were used from a wave as close in time as possible. Again, the IRT model parameters were calibrated using individuals with complete data on all tests, after which person scores were estimated for all. In this way, person scores for individuals with data from different tests were based on more items than person scores for individuals that had one or more waves on only one test.

An exception to this treatment was the QIMR data sets. There we had both the complete NEO-PI-R and the shorter NEO-FFI. Preference was always for the NEO-PI-R item data. In addition, in the QIMR data sets a distinction was made between an adolescent data set with JEPQ and NEO item data, and an adult data set with NEO, TCI, EPQ and MPQ data. Inclusion criterion for the adolescent data set was to have either JEPQ data or NEO data from waves 1, 2, or 3. In the adult data set, there were individuals with complete data on all tests. The models were calibrated on those that had complete NEO-PI-R data plus a subset of 5 000 randomly selected individuals from those that had data on at least 21 items, not being NEO items.

In case of missing item data, IRT scores were only estimated for those individuals for which there were either at least 4 dichotomous items, or at least 2 items with more than two response categories available.

*Assessing the suitability to combine tests within cohorts*

In order to assess the effect of linking two scales, correlations were computed between score estimates for item data under two calibrations: one for the items of test A and one for items of test A and B. For example, scores were estimated based on NEO Neuroticism items and a calibration that is only based on EPQ items, and then scores were also estimated using the calibration based on both EPQ and NEO items. If the EPQ and the NEO measure exactly the same trait, item parameters should not change once NEO items are included in the model calibration. Identical item parameters then result in identical score estimates based on the same item data set. Data from individuals with complete data on two tests A and B were used to calibrate a model for items from only test A, and to calibrate a model for all items. Next, the item data for only test A were used to estimate person scores based on the test A only calibration, and to estimate person scores based on the combined calibration, by assuming the B items missing at random.

**Recommendations**

Recommendations for future data harmonization projects:

1. Based on existing literature, choose the instruments that measure the target trait and that have been shown to correlate among each other.
2. Within a sample, check the fit of the IRT model to the item data for the instruments separately. Only do this for those individuals that have complete data. Check if there are any items that show misfit and that should not be included in the model. Also check your results against existing literature.
3. Within a sample, check the fit of an IRT model where item data from various instruments are combined. Preferably only do this for those individuals that have complete data on all items. Check that all discrimination parameters are positive.
4. If the model shows good fit, use the model parameters to estimate scores for all persons in the sample.
5. To check for the quality of the linking, compare the scores based on the single instrument and the combined scale as shown in section *Assessing the appropriateness to combine Neuroticism and Extraversion scores*. As a general guideline, correlations should be above 0.95.
6. Test for measurement invariance by correlating score estimates based on the calibration from one cohort to the estimates based on the calibration of a different cohort, see section *Assessing the appropriateness to combine Neuroticism and Extraversion scores*. Correlations should preferably be >0.95. Correlations lower than 0.95 might indicate potential qualitative phenotypic differences across cohort, for example general population studies versus selected studies (e.g. patients), cultural or language differences across countries, or large age or cohort differences.
7. Optionally apply hierarchical Bayesian modeling to identify variability in item parameters across studies using the Bayesian hierarchical approach (Verhagen and Fox 2012; Verhagen and Fox 2013).
8. Check the estimated scores: correlate with sum scores, and estimate familial correlations if applicable. IRT scores should show high correlations (>0.90) with sum scores (for individuals with the same item set) and twin correlations should be very similar to twin correlations based on sum scores.

**Supplementary Table 1. Overview of studies**

| Sample | Type of sample | Total number of subjects | Number of subjects included in this study | Mean age (SD)* | % of women* | Personality inventory (number of times assessed) | Year(s) of assessment |
| --- | --- | --- | --- | --- | --- | --- | --- |
| **1. ALSPAC** | Population-based  Longitudinal | 14 062 | 6 076 | 13.8 (0.21) | 51.0 | IPIP (1) | 2005-2006 |
| **2. BLSA** | Population-based  Longitudinal | 1 917 | 1 917 | 58.3 (16.6) | 49.7 | NEO-PI-R (1) | 1991-2010 |
| **3. CILENTO** | Population-based  Isolated population | 2 137 | 800 | 54.6 (19) | 64.4 | NEO-PI-R (1) | 2009-2011 |
| **4. COGEND** | Case-control study Nicotine Dependence | 2 712 | 2 712 | 36.6 (5.6) | 61.9 | NEO-FFI (1) | 2003-2007 |
| **5. EGCUT** | Population-based | 38 000 | 600 | 42.8 (16.5) | 57.3 | NEO-PI-3 (1) | 2009-2012 |
| **6. ERF** | Population-based  Isolated population | 3 000 | 2 400 | 49.3 (14.9) | 55.8 | NEO-FFI (1) | ? |
| **7. FINNISH TWINS** | Population-based  Birth cohorts  Longitudinal  Twins | 30 654 | 28 767 | 36.4 (14.60) | 50.5 | EPI (2)  NEO-FFI (1) | 1975, 1981  2003-2009 |
| **8. HBCS** | Population-based  Birth cohort  Longitudinal | 8 760 | 1 698 | 63.4 (2.9) | 55.9 | NEO-PI-R (1)  TCI (1) | 2004 |
| **9. KORCULA** | Population-based | 969 | 810 | 55.4 (13.3) | 63.1 | EPQ-R | 2007 |
| **10. LBC1921** | Population-based  Birth cohort | 498 | 478 | 81.2 (0.3) | 59.2 | IPIP (2) | 2002-2008 |
| **11. LBC1936** | Population-based  Birth cohort | 1 091 | 1 032 | 66.4 (13.5) | 50.2 | NEO-FFI (1)  IPIP (2) | 2004-2010 |
| **12. MCTFR** | Population-based  Twins | 2 232 | 2 229 | 33.4 (15.1) | 54.7 | MPQ (3) | 1998-2010 |
| **13. NBS** | Population-based | 1 823 | 1 823 | 61.5 (10.3) | 50.5 | EPQ-R (1) | 2000 |
| **14. NESDA** | Case-control study depression and anxiety  Longitudinal | 2 981 | 2 961 | 41.9 (13.1) | 66.8 | NEO-FFI | 2004-2009 |
| **15. NTR** | Population-based  Longitudinal  Twins and family members | 31 694 | 31 259 | 37.2 (15.3) | 58.7 | NEO-FFI (2)  ABV (5) | 1991-2012 |
| **16. ORCADES** | Population-based  Isolated population | 602 | 602 | 56.8 (13.8) | 57.6 | EPQ-R (1) | 2007-2011 |
| **17. PAGES** | Population-based  Healthy controls | 2 420 | 476 | 465.9 (15.4) | 55.7 | NEO-PI-R (1)  TCI (1) | 1998-2006 |
| **18. QIMR**  **adolescents** | Population-based  Longitudinal  Twins | 4 100 | 4 100 | 14.4 (2.4) | 51.5 | NEO-PI-R (1)  NEO-FFI (2)  JEPQ (3) | 1992-2011 |
| **19. QIMR**  **adults** | Population-based  Longitudinal  Twins and family members | 26 698 | 26 698 | 40.1 (15.0) | 57.1 | NEO-PI-R (1)  NEO-FFI (2)  EPQ-R (4)  TCI (1)  MPQ (1) | 1988-2007 |
| **20. SAGE-COGA** | Case-control study Alcoholism | 649 | 649 | 40.8 (10.8) | 45.6 | TCI | 1991-1998 |
| **21. STR** | Population-based  Longitudinal  Twins | 36 535 | 30 276 | 28.7 (9.1) | 52.3 | EPI | 1972 |
| **22. VIS** | Population-based  Isolated population | 918 | 909 | 56.4 (15.5) | 57.8 | EPQ-R | 2003-2004 |
| **23. YOUNG FINNS** | Population-based  Longitudinal | 3 596 | 2 057 | 37.6 (5) | 58.9 | NEO-FFI | 2007 |

* Reported for number of subjects included in this study

**Supplementary Table 2.** Overview of TCI Reward Dependence items

| Item  Number | Item | **8. HBCS** | **17. PAGES** | **19. QIMR adults** | **20. SAGE-COGA** |
| --- | --- | --- | --- | --- | --- |
| **3** | **I am often moved deeply by a fine speech or poetry.** | **S** | **S** | **S** | **S** |
| 14 | I usually do things my own way - rather than giving in to the wishes of other people. | X | X | X | X |
| **21** | **I like to discuss my experiences and feelings openly with friends instead of keeping them to myself.** | **S** | **S** | **S** | **S** |
| 28 | I like to please other people as much as I can. | X | X | X | X |
| **44** | **It wouldn't bother me to be alone all the time.** | **S** | **S** |  | **S** |
| 46 | I don't care very much whether other people like me or the way I do things. |  | X |  | X |
| **55** | **I am more sentimental than most people.** |  | **S** |  | **S** |
| **68** | **I like to keep my problems to myself.** | **S** | **S** | **S** | **S** |
| 71 | I do not think it is smart to help weak people who cannot help themselves. |  | X |  | X |
| 83 | I feel it is more important to be sympathetic and understanding of other people than to be practical and tough-minded. | X | X |  | X |
| 102 | I am strongly moved by sentimental appeals (like when asked to help crippled children). | X | X | X | X |
| **117** | **I would like to have warm and close friends with me most of the time.** | **S** | **S** |  | **S** |
| **120** | **I find sad songs and movies pretty boring.** |  | **S** |  | **S** |
| 131 | Other people often think that I am too independent because I won't do what they want. | X | X |  | X |
| **143** | **My friends find it hard to know my feelings because I seldom tell them about my private thoughts.** | **S** | **S** | **S** | **S** |
| 156 | I don't go out of my way to please other people. | X | X | X | X |
| 158 | I often give in to the wishes of friends. |  | X |  | X |
| **180** | **I usually like to stay cool and detached from other people.** | **S** | **S** | **S** | **S** |
| **181** | **I am more likely to cry at a sad movie than most people.** |  | **S** |  | **S** |
| 193 | Individual rights are more important than the needs of any group. |  | X |  | X |
| **201** | **Even when I am with friends, I prefer not to "open up" very much.** | **S** | **S** | **S** | **S** |
| **210** | **People find it easy to come to me for help, sympathy, and warm understanding.** | **S** | **S** | **S** | **S** |
| 224 | I regularly take time to consider whether what I am doing is right or wrong. |  | X |  | X |
| **226** | **If I am feeling upset, I usually feel better around friends than when left alone.** | **S** | **S** |  | **S** |

X=assessed in the sample, but not selected for the analysis

**S=assessed in the sample, and selected for the analysis (in bold)**

**Supplementary Table 3.** IRT illustration: Imaginary discrimination (*a*) and difficulty (*b*) parameters for 6 items, together with expected a posteriori (EAP) score estimates and their standard error (SE) for several imaginary response patterns (A thru E). ‘?’ indicates missing data.

|  | Items | | | | | |  |  |  |
| --- | --- | --- | --- | --- | --- | --- | --- | --- | --- |
|  | 1 | 2 | 3 | 4 | 5 | 6 |  |  |  |
| *a* | 1.18 | 1.00 | 1.12 | 1.35 | 1.03 | 0.82 |  |  |  |
| *b* | 0.76 | -1.34 | -1.54 | -0.86 | 0.97 | 1.20 | Sum score | EAP score | SE |
| A | 1 | 0 | 1 | ? | ? | ? | 2 | 0.154 | 0.798 |
| B | ? | ? | ? | 1 | 1 | 0 | 2 | 0.535 | 0.792 |
| C | 1 | 0 | 1 | 1 | 1 | 0 | 4 | 0.500 | 0.684 |
| D | 0 | 0 | 1 | 1 | 1 | 0 | 3 | -0.037 | 0.668 |
| E | 1 | 1 | 1 | 0 | 0 | 1 | 4 | 0.242 | 0.675 |

**Supplementary Table 4a.** Correlations of personality scores based on NEO-FFI item data for Neuroticism (above diagonal) and Extraversion (below diagonal) using 7 different calibrations.

|  | 4. COGEND | 6. ERF | 7. FINNISH TWINS | 11. LBC1936 | 14. NESDA | 15. NTR | 23. YOUNG FINNS |
| --- | --- | --- | --- | --- | --- | --- | --- |
| 4. COGEND | - | 0.987 | 0.979 | 0.991 | 0.911 | 0.994 | 0.967 |
| 6. ERF | 0.977 | - | 0.977 | 0.979 | 0.931 | 0.985 | 0.948 |
| 7. FINNISH TWINS | 0.994 | 0.991 | - | 0.975 | 0.923 | 0.986 | 0.953 |
| 11. LBC1936 | 0.981 | 0.987 | 0.988 | - | 0.870 | 0.998 | 0.984 |
| 14. NESDA | 0.918 | 0.952 | 0.942 | 0.969 | - | 0.893 | 0.805 |
| 15. NTR | 0.973 | 0.989 | 0.982 | 0.970 | 0.929 | - | 0.979 |
| 23. YOUNG FINNS | 0.996 | 0.978 | 0.995 | 0.978 | 0.923 | 0.974 | - |

**Supplementary Table 4b.** Correlations of personality scores based on NEO-PI-R item data for Neuroticism (above diagonal) and Extraversion (below diagonal) using 7 different calibrations.

|  | 2. BLSA | 3. CILENTO | 5. EGCUT | 8. HBCS | 17. PAGES | 18. QIMR adolescents | 19. QIMR adults |
| --- | --- | --- | --- | --- | --- | --- | --- |
| 2. BLSA | - | 0.994 | 0.989 | 0.982 | 0.994 | 0.994 | 0.997 |
| 3. CILENTO | 0.991 | - | 0.985 | 0.978 | 0.991 | 0.994 | 0.992 |
| 5. EGCUT | 0.937 | 0.953 | - | 0.973 | 0.987 | 0.986 | 0.988 |
| 8. HBCS | 0.959 | 0.946 | 0.864 | - | 0.975 | 0.984 | 0.981 |
| 17. PAGES | 0.991 | 0.984 | 0.928 | 0.959 | - | 0.989 | 0.994 |
| 18. QIMR -adolescents | 0.993 | 0.989 | 0.936 | 0.944 | 0.986 | - | 0.995 |
| 19. QIMR -adults | 0.990 | 0.987 | 0.933 | 0.938 | 0.982 | 0.993 | - |

**Supplementary Table 5.** Correlations of personality scores based on EPQ item data for Neuroticism (above diagonal) and Extraversion (below diagonal) using 5 different calibrations.

|  | 9. Korcula | 13. NBS | 16. ORCADES | 19. QIMR adults | 22. VIS |
| --- | --- | --- | --- | --- | --- |
| 9. Korcula | - | 0.962 | 0.962 | 0.987 | 0.994 |
| 13. NBS | 0.978 | - | 0.984 | 0.968 | 0.939 |
| 16. ORCADES | 0.953 | 0.989 | - | 0.984 | 0.952 |
| 19. QIMR adults | 0.968 | 0.993 | 0.997 | - | 0.986 |
| 22. VIS | 0.998 | 0.983 | 0.965 | 0.978 | - |

**Supplementary Figure 3.** Overview of personality inventories and number of Neuroticism (N) and Extraversion (E) items included per cohort.


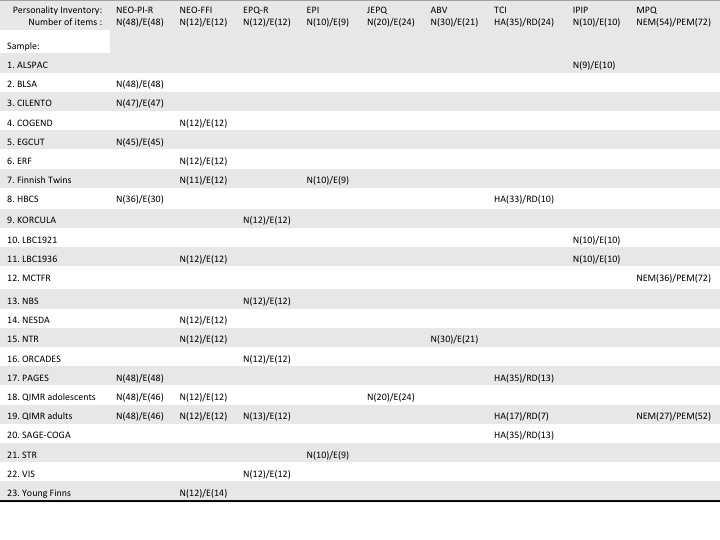


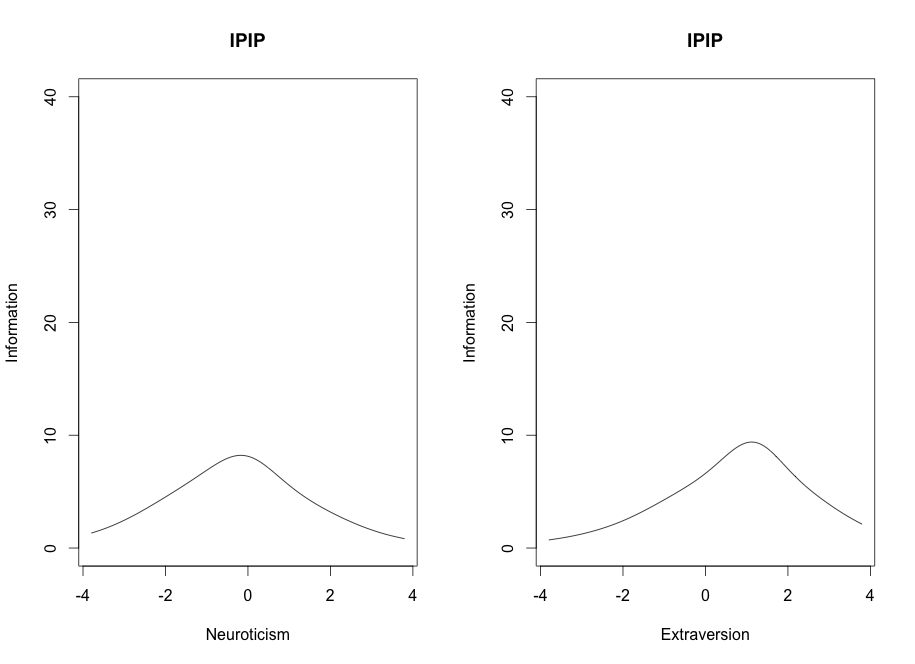


Supplementary Figure 4: Test information curves for Neuroticism and Extraversion tests in the ALSPAC cohort.


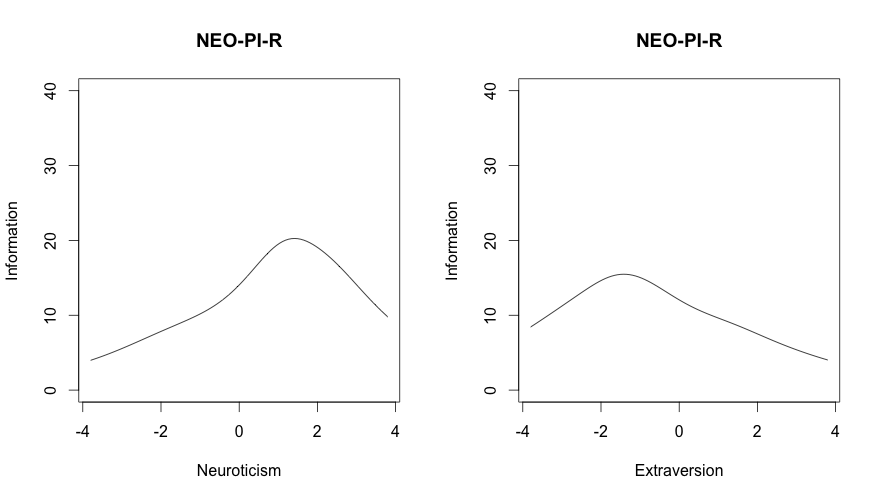


Supplementary Figure 5. Test information curves for Neuroticism and Extraversion tests in the BLSA cohort.


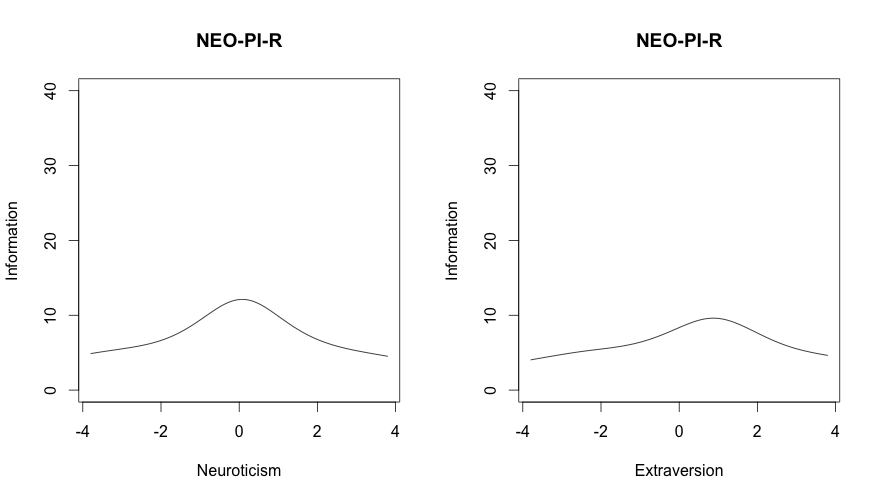


Supplementary Figure 6: Test information curves for Neuroticism and Extraversion tests in the CILENTO sample.


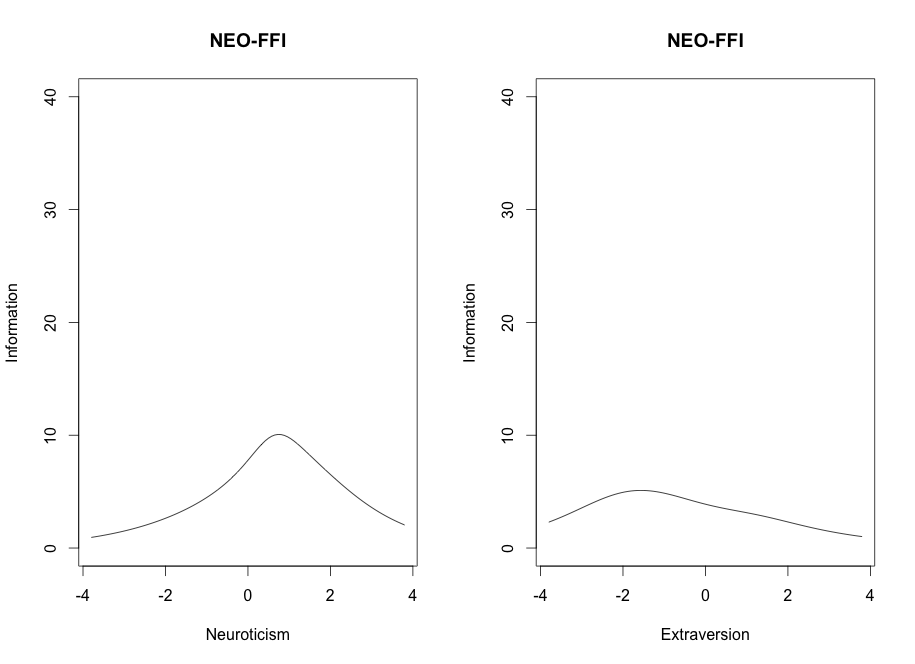


Supplementary Figure 7: Test information curves for Neuroticism and Extraversion tests in the COGEND sample.


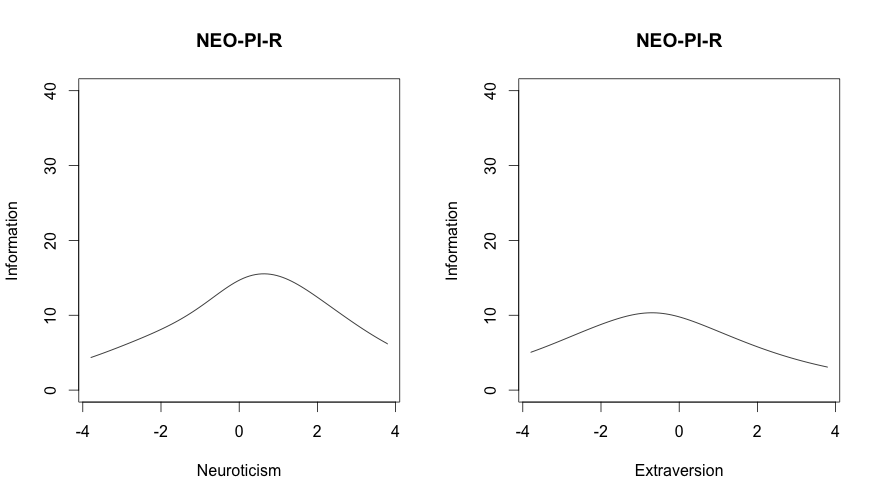


Supplementary Figure 8: Test information curves for Neuroticism and Extraversion tests in the EGCUT sample.


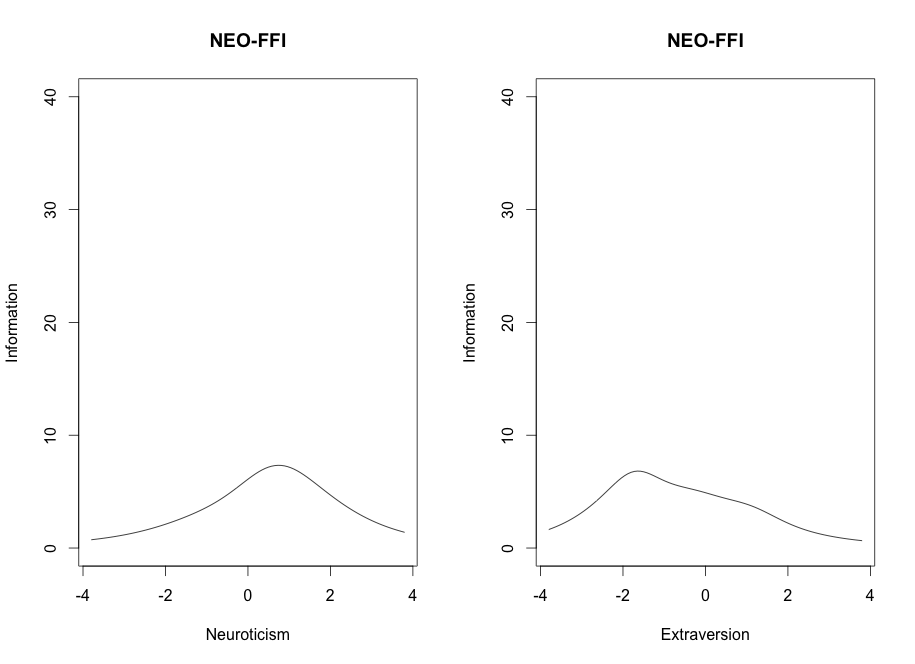


Supplementary Figure 9: Test information curves for Neuroticism and Extraversion tests in the ERF sample.


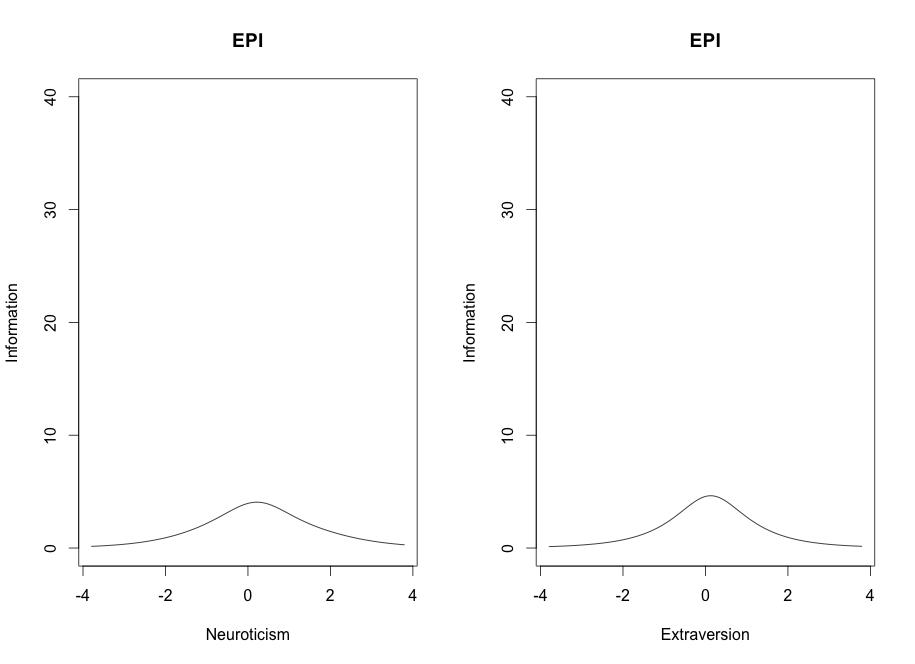


Supplementary Figure 10: Test information curves for Neuroticism and Extraversion tests in the Finnish Twins sample.


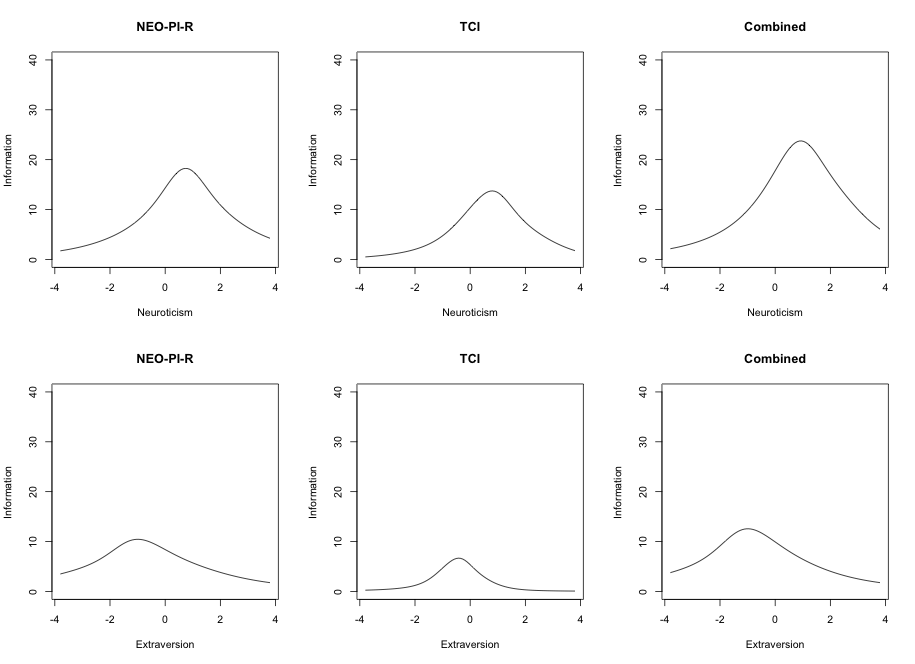


Supplementary Figure 11: Test information curves for Neuroticism and Extraversion tests in the HBCS sample.


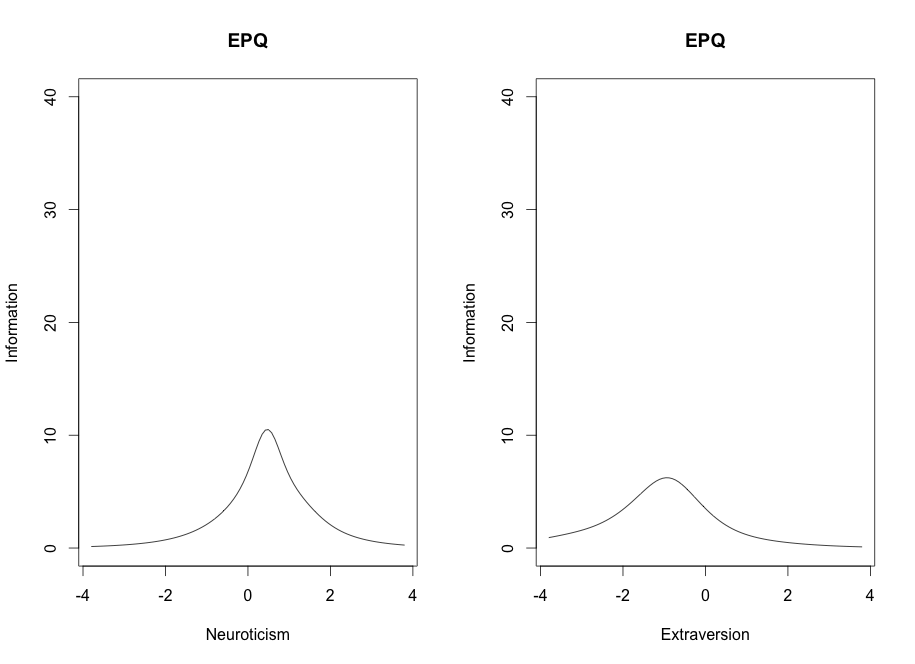


Supplementary Figure 12: Test information curves for Neuroticism and Extraversion tests in the Korcula sample


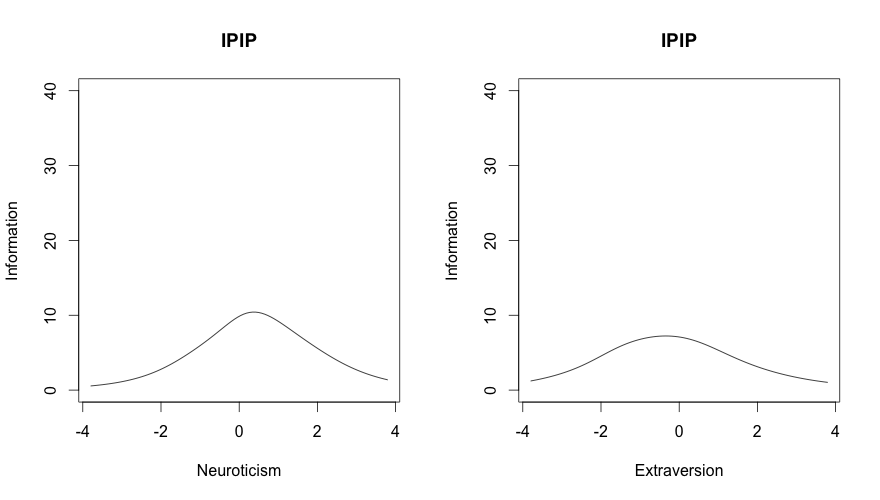


Supplementary Figure 13: Test information curves for Neuroticism and Extraversion tests in the LBC1921 sample


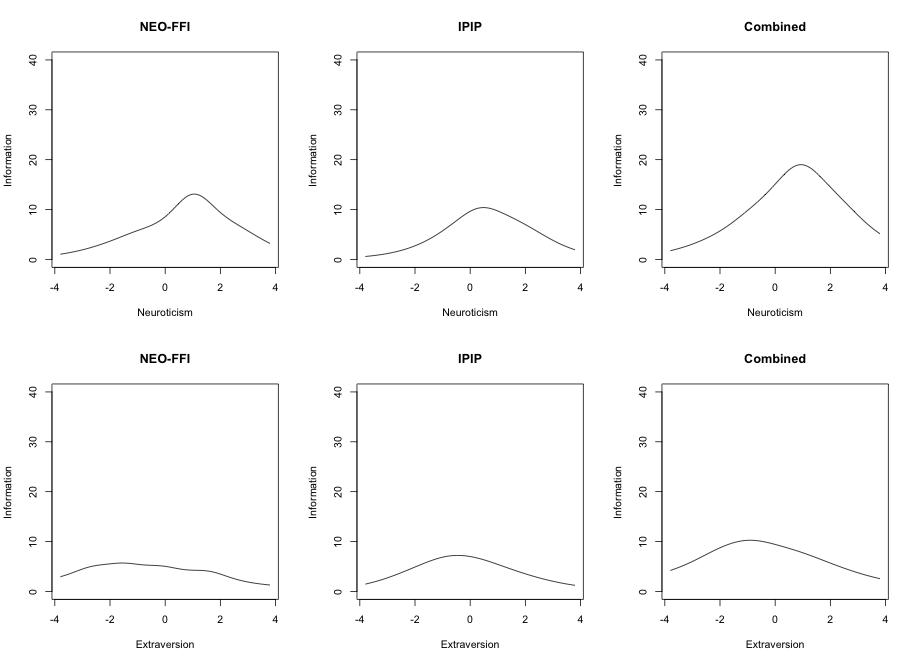


Supplementary Figure 14: Test information curves for Neuroticism and Extraversion tests in the LBC1936 sample.


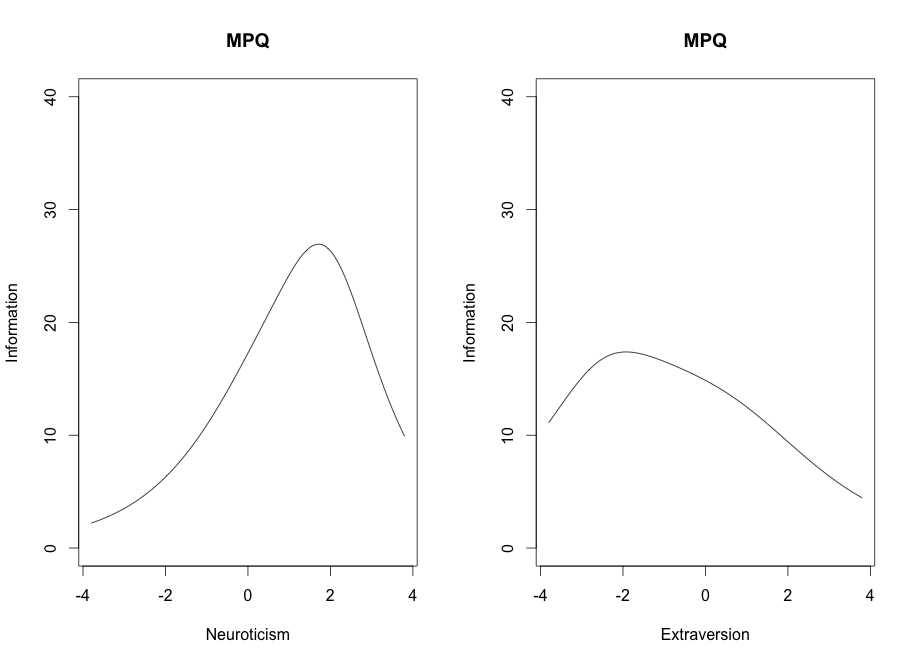


Supplementary Figure 15: Test information curves for Neuroticism and Extraversion tests in the MCTFR sample.


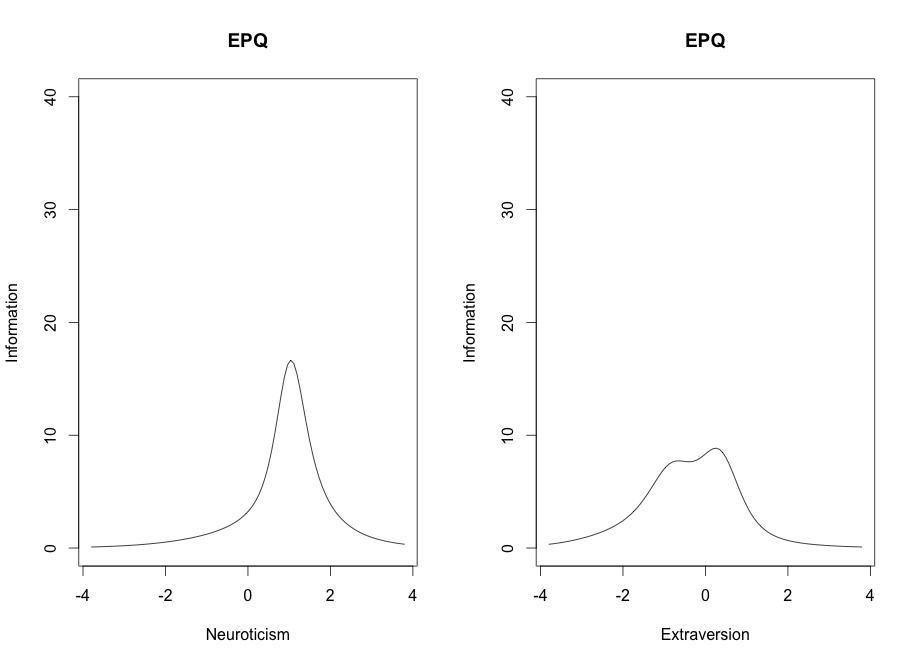


Supplementary Figure 16: Test information curves for Neuroticism and Extraversion tests in the NBS sample.


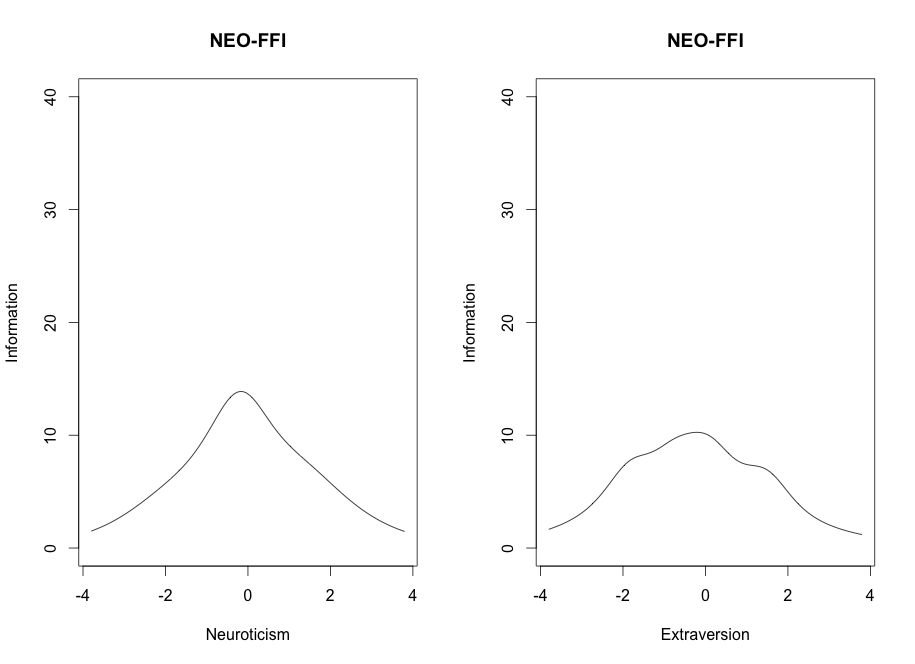


Supplementary Figure 17: Test information curves for Neuroticism and Extraversion tests in the NESDA sample.


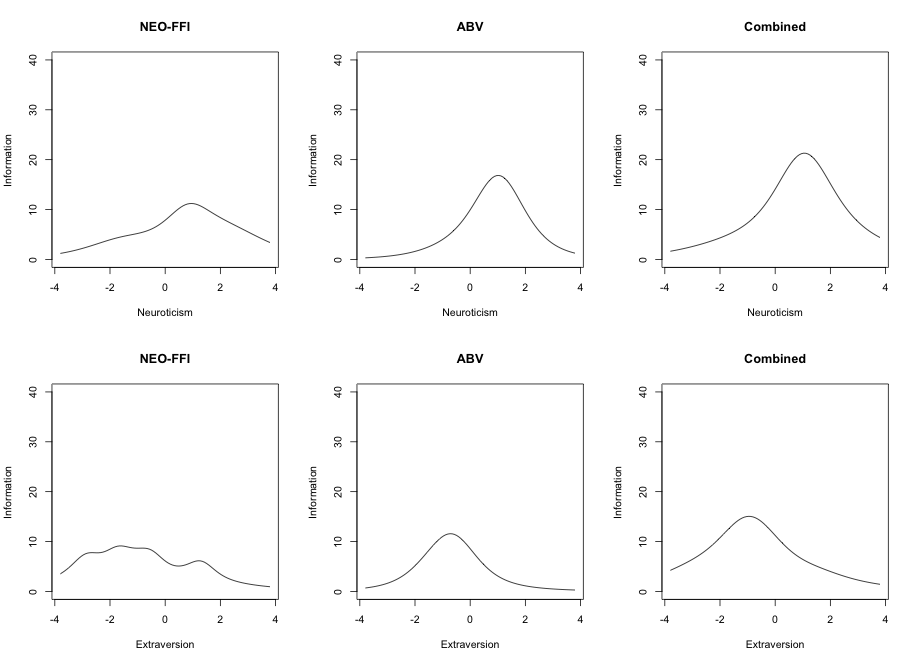


Supplementary Figure 18: Test information curves for Neuroticism and Extraversion tests in the NTR sample.


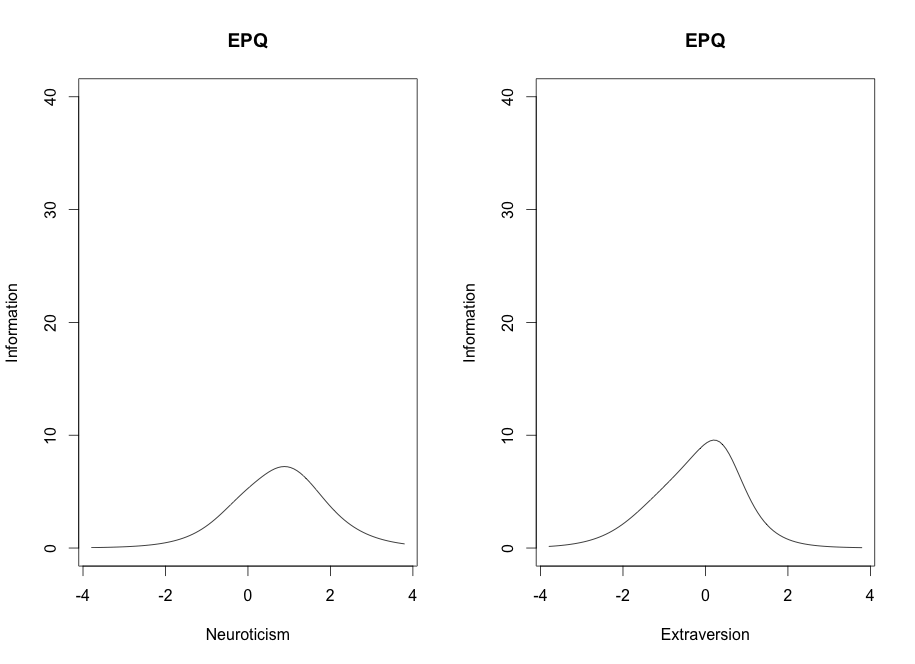


Supplementary Figure 19: Test information curves for Neuroticism and Extraversion tests in the ORCADES cohort.


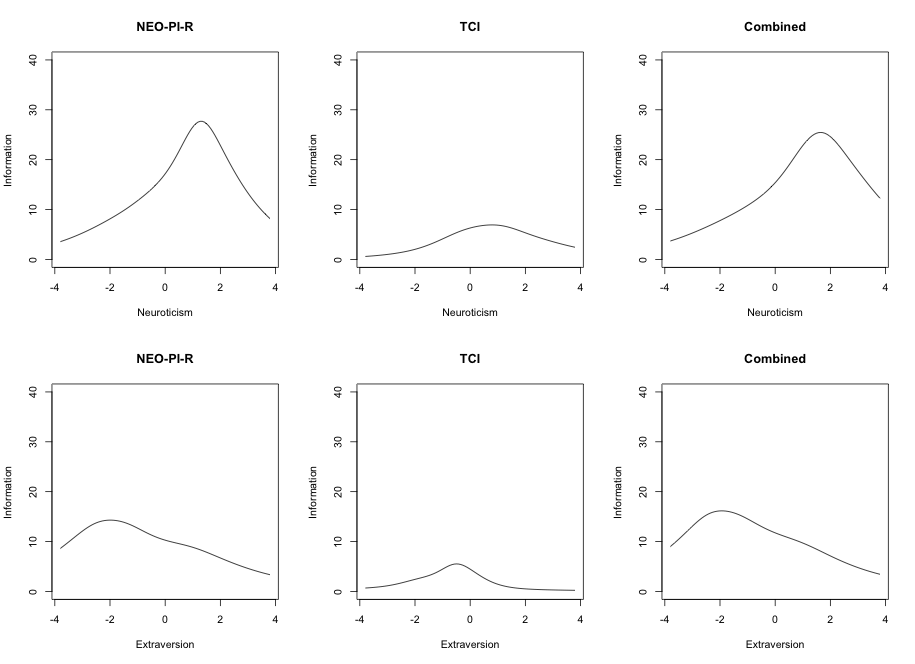


Supplementary Figure 20: Test information curves for Neuroticism and Extraversion tests in the PAGES cohort.


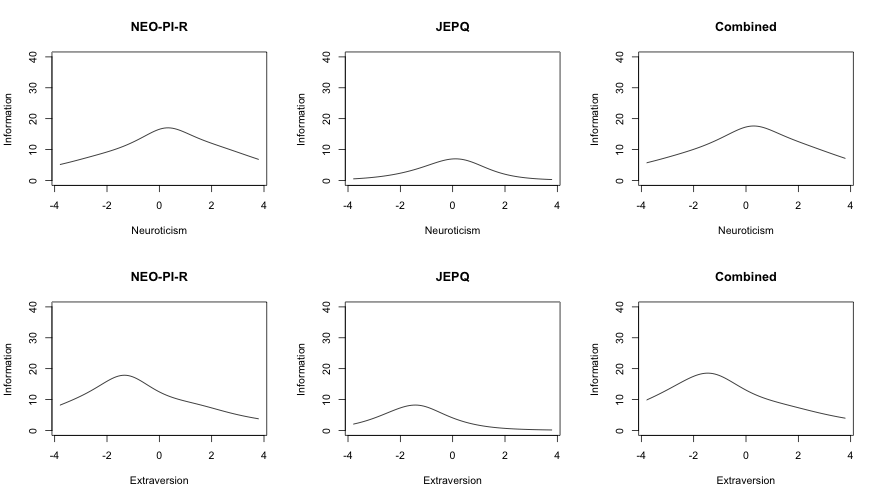


Supplementary Figure 21: Test information curves for Neuroticism and Extraversion tests in the QIMR adolescent sample.


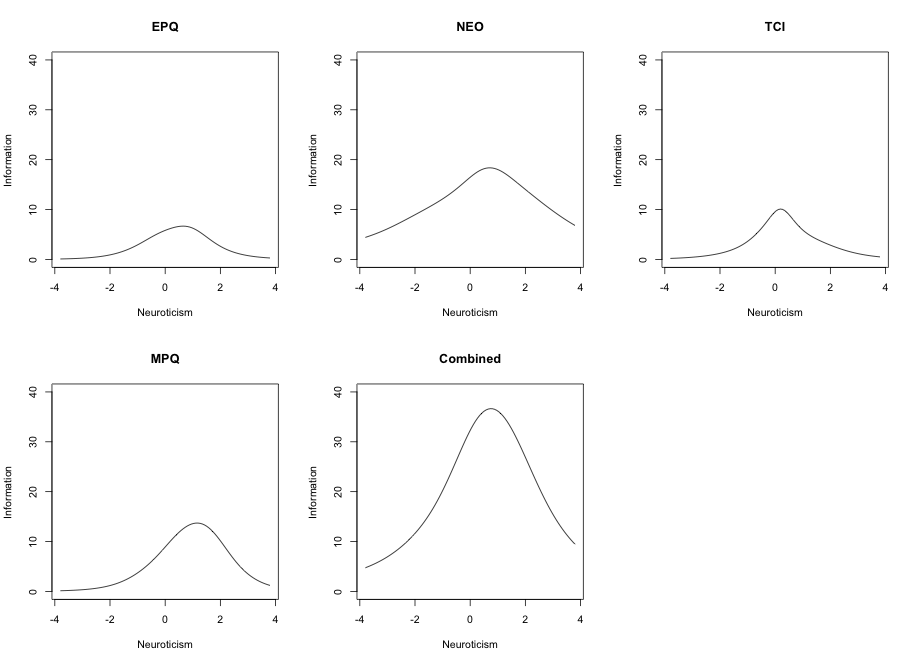


Supplementary Figure 22: Test information curves for Neuroticism tests in the QIMR adult sample.


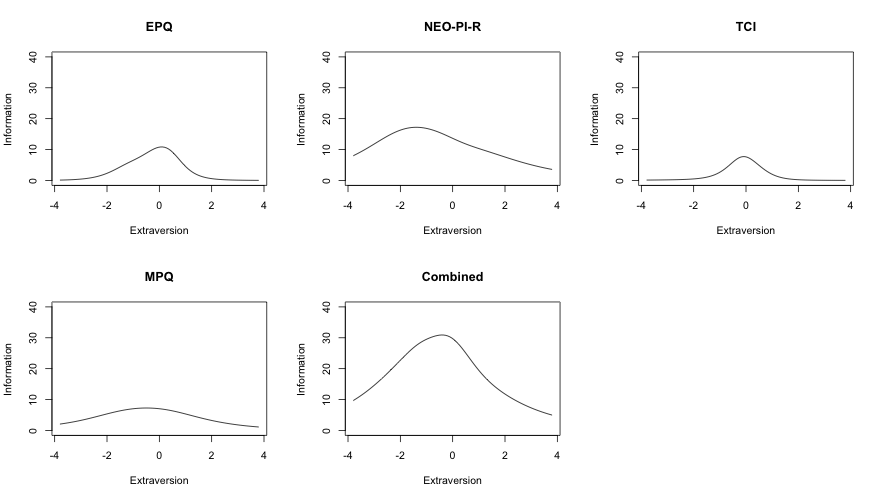


Supplementary Figure 23: Test information curves for Extraversion tests in the QIMR adult sample.


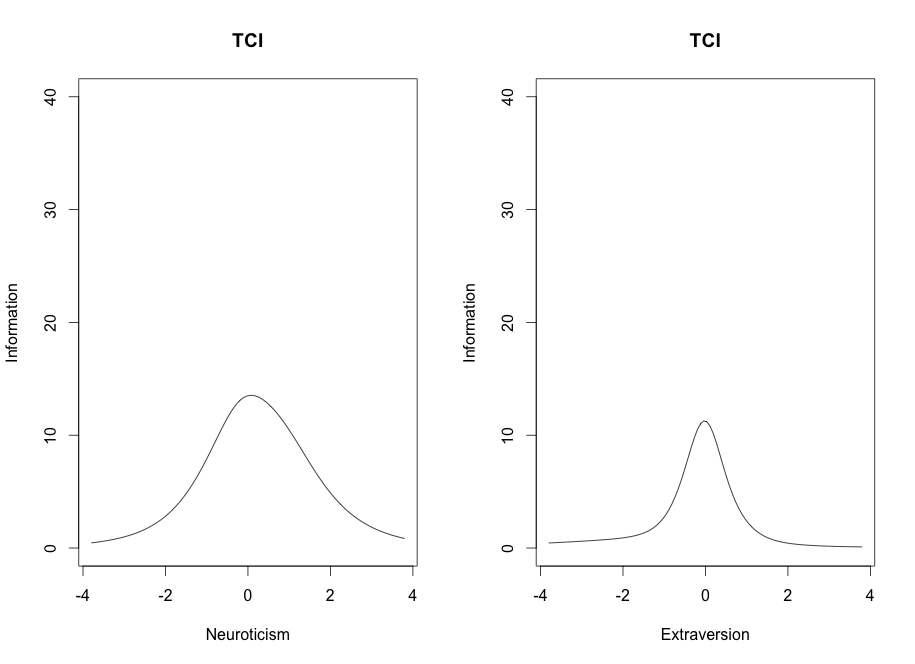


Supplementary Figure 24: Test information curves for Neuroticism and Extraversion tests in the SAGE-COGA cohort.


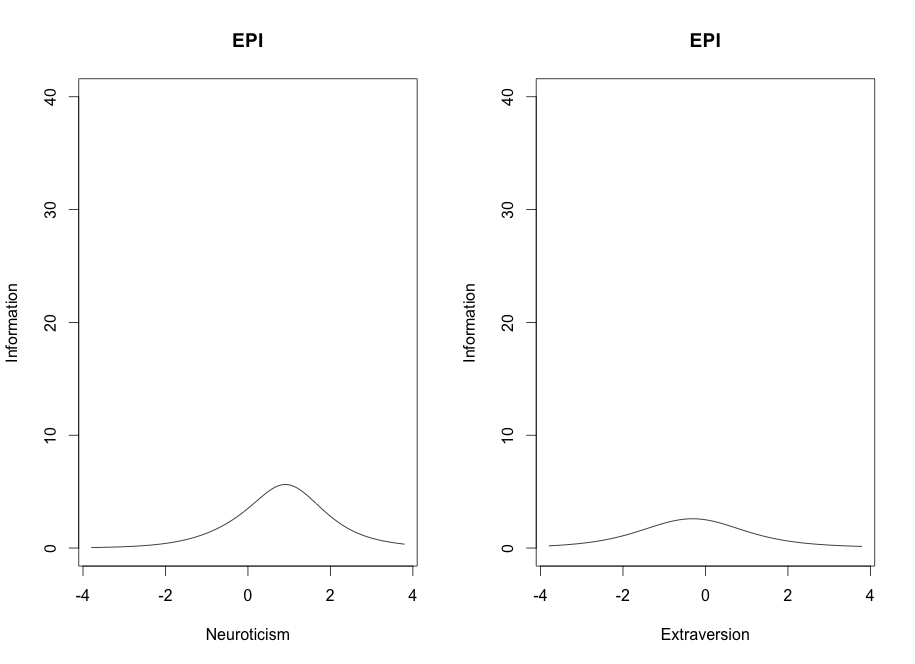


Supplementary Figure 25: Test information curves for Neuroticism and Extraversion tests in the STR cohort.


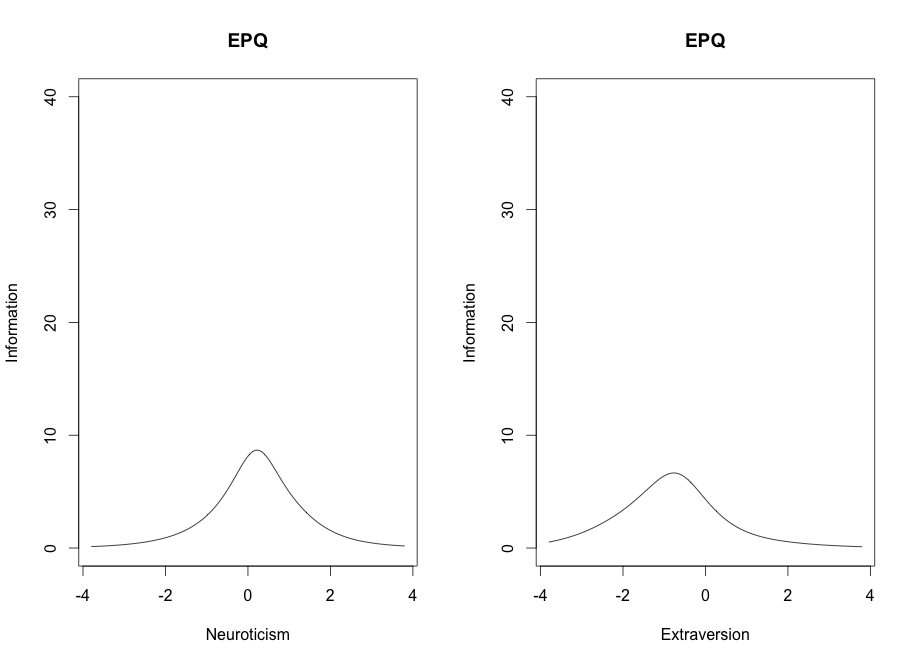


Supplementary Figure 26: Test information curves for Neuroticism and Extraversion tests in the VIS cohort


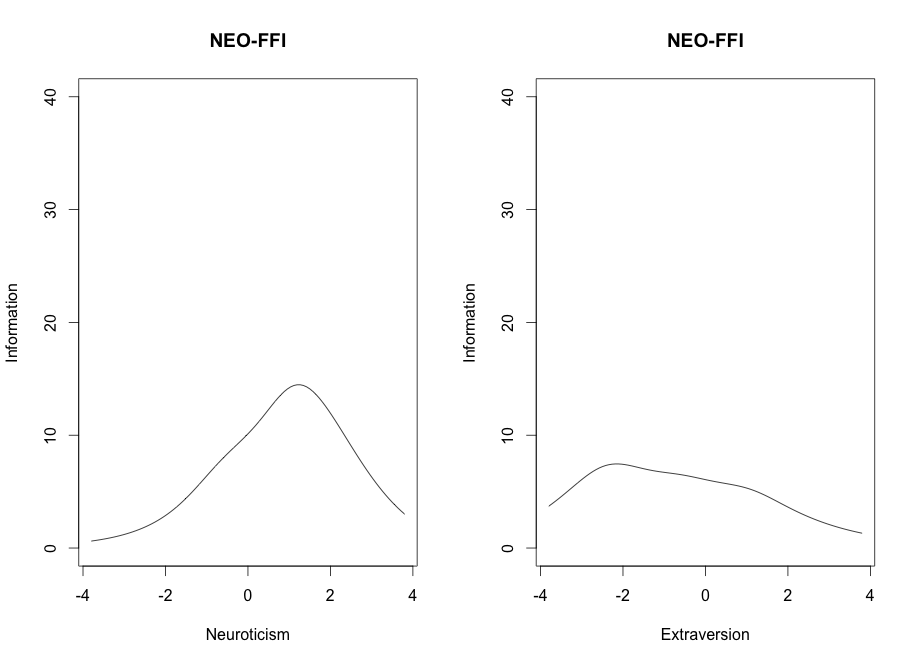


Supplementary Figure 27: Test information curves for Neuroticism and Extraversion tests in the YoungFinns cohort.

**Example R code to link two tests for one sample**

# it is assumed that the saved R object 'data_file' contains Person_ID, Family_ID, Sex and Age in columns 1-4

# and the selected items from inventory/test A (columns 5:16)

# and the selected items from inventory/test B (columns 17:28)

load(file="data_file")

library(ltm) # required for IRT analyses

# code 9 is missing item data (if applicable)

for (i in 5:(dim(data_file)[2]))

{

data_file[which(data_file[,i]==9),i] <- NA

}

# select data only from those individuals that have complete data on both tests:

data.complete.cases <- data_file[ complete.cases(data_file[,5:28]) ,5:28]

summary(data.complete.cases)

# for IRT score estimation to go well, lowest category number should be 1, rather than 0

# thus, 0/1 should be scored as 1/2 data, 0/1/2 data should be rescored as 1/2/3

data.complete.cases[,1:24] <- data.complete.cases[,1:24]+1 # to avoid problems with theta estimation

# do IRT analysis separately for test A

out.A<- gpcm(data.complete.cases[,1:12], IRT.param = T, control=list(iter.qN=600, GHk=23))

summary(out.A) # gives item parameters

# estimate scores based on test A analysis for people with complete data

factor.scores.A<- factor.scores(out.A,method = "EAP", resp.patterns=data.complete.cases[,1:12])

# do IRT analysis separately for test B

out.B<- gpcm(data.complete.cases[,13:24], IRT.param = T, control=list(iter.qN=600, GHk=23))

summary(out.B) # gives item parameters

# estimate scores based on test B analysis for people with complete data

factor.scores.B<- factor.scores(out.B,method = "EAP", resp.patterns=data.complete.cases[,13:24])

# do IRT analysis separately for tests A and B together:

out.AB<- gpcm(data.complete.cases[,1:24], IRT.param = T, control=list(iter.qN=600, GHk=23))

summary(out.AB) # gives item parameters

# estimate scores based on tests A and B analysis for people with complete data

factor.scores.AB<- factor.scores(out.AB,method = "EAP", resp.patterns=data.complete.cases[,1:24])

# good idea to save IRT results if analysis took a while:

save.image("example_analysis.RData")

load("example_analysis.RData ")

# correlation between estimated scores on tests A and B

cor((factor.scores.A$score.dat)$z1, (factor.scores.B$score.dat)$z1 )

plot((factor.scores.A$score.dat)$z1, (factor.scores.B$score.dat)$z1,xlab='Test A score',ylab='Test B score' )

cor((factor.scores.AB$score.dat)$z1, (factor.scores.A$score.dat)$z1 )

plot((factor.scores.AB$score.dat)$z1, (factor.scores.A$score.dat)$z1,xlab='Tests A and B score',ylab='Test A score' )

cor((factor.scores.AB$score.dat)$z1, (factor.scores.B$score.dat)$z1 )

plot((factor.scores.AB$score.dat)$z1, (factor.scores.B$score.dat)$z1,xlab='Tests A and B score',ylab='Test B score' )

# model fit: parameter values should not be much affected by adding extra items from other test to the analysis

# dots should therefore be on straight lines

par(mfrow = c(1, 2))

plot(unlist(out.A$coef[1:12]),unlist(out.AB$coef[1:12]),ylab="Parameter values when A and B combined", xlab="Parameter values A")

plot( unlist(out.B$coef[1:12]), unlist(out.AB$coef[13:24]),ylab="Parameter values A and B combined", xlab="Parameter values B")

# model fit: how much do item parameters change once test data from other inventory is added to the analysis?

# ideally, this should not affect item parameters at all, and therefore not affect ordering of individuels

A <- cbind(data.complete.cases[,1:12],matrix(NA, length(data.complete.cases[,1]),12))

A_combined<- factor.scores(out.AB,method = "EAP",resp.patterns =A[,1:24])

plot((factor.scores.A$score.dat)$z1, (A_combined$score.dat)$z1,xlab='Test A score',ylab='Test A B score if only A data were used') # should be close to straight line

cor((factor.scores.A$score.dat)$z1, (A_combined$score.dat)$z1) # should be close to 1

B<- cbind(matrix(NA, length(data.complete.cases[,1]),12),data.complete.cases[,13:24])

B_combined<- factor.scores(out.AB,method = "EAP",resp.patterns =B[,1:24])

plot((factor.scores.B$score.dat)$z1, (B_combined$score.dat)$z1, xlab='Test B score',ylab='Test A B score if only B data were used')

cor((factor.scores.B$score.dat)$z1, (B_combined$score.dat)$z1)

# Model fit: Plot test information functions

# Ideally, the combined information function should be the exact sum of the

# information functions of tests A and B separately

par(mfrow = c(1, 3))

plot(out.A, type = c("IIC"), items = 0, ylim=c(0,20), main="Test A", xlab='Latent score')

plot(out.B, type = c("IIC"), items = 0, ylim=c(0,20), main="Test B", xlab='Latent score')

plot(out.AB, type = c("IIC"), items = 0, ylim=c(0,20), main="Tests A and B combined", xlab='Latent score')

# compute unweighted sumscores:

A.sum<- apply(data.complete.cases[,1:12],1,sum)

B.sum<- apply(data.complete.cases[,13:24],1,sum)

#

plot(A.sum,B.sum, xlab='Test A sum score', ylab='Test B sum score')

cor(A.sum,B.sum)

# sum scores should correlate highly with IRT based estimates,

# exactly how high depends on how different the discrimination parameters are

# if discrimination parameters are very similar, correlation is higher

# plot usually shows an S-curve

cor(A.sum, (factor.scores.A$score.dat)$z1)

plot(A.sum, (factor.scores.A$score.dat)$z1, xlab='Sum score test A', ylab='IRT score test A')

cor(B.sum, (factor.scores.B$score.dat)$z1)

plot(B.sum, (factor.scores.B$score.dat)$z1, xlab='Sum score test B', ylab='IRT score test B')

# if everything above looks OK, IRT scores can be estimated for all indvidiuals, including those with missing data

# Now getting all data, and compute factor scores based on calibrated IRT model

raw.data<- (data_file[, 5:28])

raw.data<- raw.data+1 # if needed, see above

# estimate scores based on the calibration using all items from tests A and B

factor.scores<- factor.scores(out.AB,resp.patterns=raw.data, method="EAP")

# how many persons have how many items?

table(apply(raw.data, 1, function(x) sum(is.na(x)==F)))

data_file[apply(raw.data, 1, function(x) sum(is.na(x)==F))==0,1:3] # plot individuals with no data

# give these individuals with no data, a missing value for the estimate

(factor.scores$score.dat)$z1[which(apply(raw.data, 1, function(x) sum(is.na(x)==F))==0)] <- NA

# save your results:

thetas<- cbind(data_file[,1:4],round((factor.scores$score.dat)$z1,3),round((factor.scores$score.dat)$se.z1,3),apply(raw.data, 1, function(x) sum(is.na(x)==F)) )

# theta is the estimated IRT-based score, se.theta is the standard error of measurement for that estimate

colnames(thetas)<- c("Pers_ID", "Fam_ID", "Sex", 'Age',"theta", "se.theta", "N.items")

write.table(thetas, file="thetas.dat", row.names=F)

write.csv(thetas, file="thetas.csv", row.names=F)

write.csv2(thetas, file="thetas2.csv", row.names=F)

References

Aitken JF, Green A, Eldridge A, Green L, Pfitzner J, Battistutta D, Martin NG (1994) Comparability of Nevus Counts Between and Within Examiners, and Comparison with Computer Image-Analysis. British Journal of Cancer 69(3):487-491

Allik J, Laidra K, Realo A, Pullmann H (2004) Personality development from 12 to 18 years of age: Changes in mean levels and structure of traits. European Journal of Personality 18(6):445-462

Aluja A, Garcia O, Garcia LF (2004) Replicability of the three, four and five Zuckerman's personality super-factors: exploratory and confirmatory factor analysis of the EPQ-RS, ZKPQ and NEO-PI-R. Personality and Individual Differences 36(5):1093-1108

Barker DJP, Osmond C, Forsen TJ, Kajantie E, Eriksson JG (2005) Trajectories of growth among children who have coronary events as adults. New England Journal of Medicine 353(17):1802-1809

Bierut LJ, Madden PAF, Breslau N, Johnson EO, Hatsukami D, Pomerleau OF, Swan GE, Rutter J, Bertelsen S, Fox L, Fugman D, Goate AM, Hinrichs AL, Konvicka K, Martin NG, Montgomery GW, Saccone NL, Saccone SF, Wang JC, Chase GA, Rice JP, Ballinger DG (2007) Novel genes identified in a high-density genome wide association study for nicotine dependence. Human Molecular Genetics 16(1):24-35

Bock RD, Mislevy RJ (1982) Adaptive Eap Estimation of Ability in a Microcomputer Environment. Appl Psych Meas 6(4):431-444

Boomsma DI, de Geus EJC, Vink JM, Stubbe JH, Distel MA, Hottenga JJ, Posthuma D, Beijsterveldt TCEM, Hudziak JJ, Bartels M, Willemsen G (2006) Netherlands Twin Register: From twins to twin families. Twin Research and Human Genetics 9(6):849-857

Boomsma DI, Vink JM, van Beijsterveldt TC, de Geus EJ, Beem AL, Mulder EJ, Derks EM, Riese H, Willemsen GA, Bartels M, van den BM, Kupper NH, Polderman TJ, Posthuma D, Rietveld MJ, Stubbe JH, Knol LI, Stroet T, van Baal GC (2002) Netherlands Twin Register: a focus on longitudinal research. Twin Research 5(5):401-406

Boyd A, Golding J, Macleod J, Lawlor DA, Fraser A, Henderson J, Molloy L, Ness A, Ring S, Davey Smith G (2013) Cohort Profile: The ‘Children of the 90s’—the index offspring of the Avon Longitudinal Study of Parents and Children. International Journal of Epidemiology 42(1):111-127

Cloninger C SDMPTR (1993) A psychobiological model of temperament and character. Archives of General Psychiatry 50(12):975-990

Colonna V, Nutile T, Astore M, Guardiola O, Antoniol G, Ciullo M, Persico MG (2007) Campora: A young genetic isolate in South Italy. Hum Hered 64(2):123-135

Colonna V, Nutile T, Ferrucci RR, Fardella G, Aversano M, Barbujani G, Ciullo M (2009) Comparing population structure as inferred from genealogical versus genetic information. European Journal of Human Genetics 17(12):1635-1641

Costa PT, McCrae RR (1992) Professional manual: Revised NEO Personality Inventory (NEO-PI-R) and NEO Five-Factor- Inventory (NEO-FFI). Psychological Assessment Resources, Odessa, FL

De Fruyt F, Van de Wiele L, Van Heeringen C (2000) Cloninger's psychobiological model of temperament and character and the five-factor model of personality. Personality and Individual Differences 29(3):441-452

Deary IJ, Gow AJ, Pattie A, Starr JM (2011) Cohort Profile: The Lothian Birth Cohorts of 1921 and 1936. International Journal of Epidemiology Epub ahead of print

Deary IJ, Gow AJ, Taylor MD, Corley J, Brett C, Wilson V, Campbell H, Whalley LJ, Visscher PM, Porteous DJ, Starr JM (2007) The Lothian Birth Cohort 1936: a study to examine influences on cognitive ageing from age 11 to age 70 and beyond. BMC Geriatrics 728

Deary IJ, Whiteman MC, Starr JM, Whalley LJ, Fox HC (2004) The impact of childhood intelligence on later life: Following up the Scottish Mental Surveys of 1932 and 1947. J Pers Soc Psychol 86(1):130-147

Distel MA, Trull TJ, Derom CA, Thiery EW, Grimmer MA, Martin NG, Willemsen G, Boomsma DI (2008) Heritability of borderline personality disorder features is similar across three countries. Psychol Med 38(9):1219-1229

Draycott SG, Kline P (1995) The Big-3 Or the Big-5 - the Epq-R Vs the Neo-Pi - A Research Note, Replication and Elaboration. Personality and Individual Differences 18(6):801-804

Eriksson JG, Osmond C, Kajantie E, Forsen TJ, Barker DJP (2006) Patterns of growth among children who later develop type 2 diabetes or its risk factors. Diabetologia 49(12):2853-2858

Eysenck HJ, Eysenck SBG (1964) Eysenck Personality Inventory. Educational and Industrial Testing Service, San Diego, CA

Eysenck HJ, Eysenck SBG (1975) Manual of the Eysenck Personality Questionnaire, London: Hodder & Stoughton

Eysenck SBG (1972) Junior Eysenck Personality Inventory. EdITS/Educational and Industrial Testing Service, San Diego (CA)

Eysenck SBG, Eysenck HJ, Barrett P (1985) A Revised Version of the Psychoticism Scale. Personality and Individual Differences 6(1):21-29

Floderus-Myrhed B, Pedersen N, Rasmuson I (1980) Assessment of heritability for personality, based on a short-form of the Eysenck Personality Inventory: a study of 12 898 twin pairs. Behavior Genetics 10153-162

Foroud T, Edenberg HJ, Goate A, Rice J, Flury L, Koller DL, Bierut LJ, Conneally PM, Nurnberger JI, Bucholz KK, Li TK, Hesselbrock V, Crowe R, Schuckit M, Porjesz B, Begleiter H, Reich T (2000) Alcoholism susceptibility loci: Confirmation studies in a replicate sample and further mapping. Alcoholism-Clinical and Experimental Research 24(7):933-945

Gillespie NA, Johnstone SJ, Boyce P, Heath AC, Martin NG (2001) The genetic and environmental relationship between the interpersonal sensitivity measure (IPSM) and the personality dimensions of Eysenck and Cloninger. Personality and Individual Differences 31(7):1039-1051

Goldberg LR (1999) A broad-bandwidth, public-domain, personality inventory measuring the lower-level facets of several Five-Factor models. In: Mervielde I, Deary IJ, De Fruyt F (eds) Personality psychology in Europe. Tilburg University Press, Tilburg, pp 7-28

Hansell NK, Agrawal A, Whitfield JB, Morley KI, Zhu G, Lind PA, Pergadia ML, Madden PAF, Todd RD, Heath AC, Martin NG (2008) Long-term stability and heritability of telephone interview measures of alcohol consumption and dependence. Twin Research and Human Genetics 11(3):287-305

Heath AC, Jardine R, Eaves LJ, Martin NG (1988) The genetic structure of personality I. Phenotypic factor structure of the EPQ in an Australian sample. Personality and Individual Differences 9(1):59-67

Iacono WG, McGue M (2002) Minnesota Twin Family Study. Twin Research 5(5):482-487

Ivkovic V, Vitart V, Rudan I, Janicijevic B, Smolej-Narancic N, Skaric-Juric T, Barbalic M, Polasek O, Kolcic I, Biloglav Z, Visscher PM, Hayward C, Hastie ND, Anderson N, Campbell H, Wright AF, Rudan P, Deary IJ (2007) The Eysenck personality factors: Psychometric structure, reliability, heritability and phenotypic and genetic correlations with psychological distress in an isolated Croatian population. Personality and Individual Differences 42(1):123-133

Kallasmaa T, Allik J, Realo A, McCrae RR (2000) The Estonian version of the NEO-PI-R: An examination of universal and culture-specific aspects of the five-factor model. European Journal of Personality 14(3):265-278

Kaprio J (2006) Twin studies in Finland 2006. Twin Research and Human Genetics 9(6):772-777

Kaprio J (2013) The Finnish Twin Cohort Study: An Update. Twin Research and Human Genetics 16(Special Issue 01):157-162

Kiemeney LA, Thorlacius S, Sulem P, Geller F, Aben KKH, Stacey SN, Gudmundsson J, Jakobsdottir M, Bergthorsson JT, Sigurdsson A, Blondal T, Witjes JA, Vermeulen SH, Hulsbergen-Van De Kaa CA, Swinkels DW, Ploeg M, Cornel EB, Vergunst H, Thorgeirsson TE, Gudbjartsson D, Gudjonsson SA, Thorleifsson G, Kristinsson KT, Mouy M, Snorradottir S, Placidi D, Campagna M, Arici C, Koppova K, Gurzau E, Rudnai P, Kellen E, Polidoro S, Guarrera S, Sacerdote C, Sanchez M, Saez B, Valdivia G, Ryk C, De Verdier P, Lindblom A, Golka K, Bishop DT, Knowles MA, Nikulasson S, Petursdottir V, Jonsson E, Geirsson G, Kristjansson B, Mayordomo JI, Steineck G, Porru S, Buntinx F, Zeegers MP, Fletcher T, Kumar R, Matullo G, Vineis P, Kiltie AE, Gulcher JR, Thorsteinsdottir U, Kong A, Rafnar T, Stefansson K (2008) Sequence variant on 8q24 confers susceptibility to urinary bladder cancer. Nature Genetics 40(11):1307-1312

Kirk KM, Birley AJ, Stratham DJ, Haddon B, Lake RIE, Andrews JG, Martin NG (2000) Anxiety and depression in twin and sib pairs extremely discordant and concordant for neuroticism: prodromus to a linkage study. Twin Research 3299-309

Larstone RM, Jang KL, Livesley WJ, Vernon PA, Wolf H (2002) The relationship between Eysenck's P-E-N model of personality, the five-factor model of personality, and traits delineating personality dysfunction. Personality and Individual Differences 33(1):25-37

Little RJA, Rubin DB (1989) The analysis of social science data with missing values. Sociological Methods & Research 18292-326

Lord FM (1980) Applications of item response theory to practical testing problems. Erlbaum, Mahwah, NJ

McCrae RR, Costa PT (2004) A contemplated revision of the NEO Five-Factor Inventory. Personality and Individual Differences 36(3):587-596

McCrae RR, Costa PT, Martin TA (2005) The NEO-PI-3: A more readable revised NEO Personality Inventory. Journal of Personality Assessment 84(3):261-270

McGue M, Keyes M, Sharma A, Elkins I, Legrand L, Johnson W, Iacono WG (2007) The environments of adopted and non-adopted youth: Evidence on range restriction from the Sibling Interaction and Behavior Study (SIBS). Behavior Genetics 37(3):449-462

McQuillan R, Leutenegger AL, bdel-Rahman R, Franklin CS, Pericic M, Barac-Lauc L, Smolej-Narancic N, Janicijevic B, Polasek O, Tenesa A, MacLeod AK, Farrington SM, Rudan P, Hayward C, Vitart V, Rudan I, Wild SH, Dunlop MG, Wright AF, Campbell H, Wilson JF (2008) Runs of homozygosity in European populations. Am J Hum Genet 83(3):359-372

Metspalu A (2004) The Estonian Genome Project. Drug Development Research 62(2):97-101

Muraki E (1992) A Generalized Partial Credit Model: Application of an EM algorithm. Appl Psych Meas 16159-176

Pardo LM, MacKay I, Oostra B, van Duijn CM, Aulchenko YS (2005) The effect of genetic drift in a young genetically isolated population. Annals of Human Genetics 69288-295

Penninx BWJH, Beekman ATF, Smit JH, Zitman FG, Nolen WA, Spinhoven P, Cuijpers P, De Jong PJ, Van Marwijk HWJ, Assendelft WJJ, Van Der Meer K, Verhaak P, Wensing M, De Graaf R, Hoogendijk WJ, Ormel J, Van Dyck R, Consortium NR (2008) The Netherlands Study of Depression and Anxiety (NESDA): rationale, objectives and methods. International Journal of Methods in Psychiatric Research 17(3):121-140

Pergadia ML, Agrawal A, Loukola A, Montgomery GW, Broms U, Saccone SF (2009) Genetic linkage findings for DSM-IV nicotine withdrawal in two populations. Am J Med Genet B 150B950-959

Polasek O, Marusic A, Rotim K, Hayward C, Vitart V, Huffman J, Campbell S, Jankovic S, Boban M, Biloglav Z, Kolcic I, Krzelj V, Terzic J, Matec L, Tometic G, Nonkovic D, Nincevic J, Pehlic M, Zedelj J, Velagic V, Juricic D, Kirac I, Kovacevic SB, Wright AF, Campbell H, Rudan I (2009) Genome-wide Association Study of Anthropometric Traits in Korcula Island, Croatia. Croatian Medical Journal 50(1):7-16

Raikkonen K, Pesonen AK, Heinonen K, Lahti J, Kajantie E, Forsen T, Osmond C, Barker DJP, Eriksson JG (2008) Infant growth and hostility in adult life. Psychosomatic Medicine 70(3):306-313

Raitakari OT, Juonala M, Ronnemaa T, Keltikangas-Jarvinen L, Rasanen L, Pietikainen M, Hutri-Kahonen N, Taittonen L, Jokinen E, Marniemi J, Jula A, Telama R, Kahonen M, Lehtimaki T, Akerblom HK, Viikari JSA (2008) Cohort Profile: The Cardiovascular Risk in Young Finns Study. International Journal of Epidemiology 37(6):1220-1226

Reich T, Edenberg HJ, Goate A, Williams JT, Rice JP, Van Eerdewegh P, Foroud T, Hesselbrock V, Schuckit MA, Bucholz K, Porjesz B, Li TK, Conneally PM, Nurnberger JI, Tischfield JA, Crowe RR, Cloninger CR, Wu W, Shears S, Carr K, Crose C, Willig C, Begleiter H (1998) Genome-wide search for genes affecting the risk for alcohol dependence. American Journal of Medical Genetics 81(3):207-215

Rizopoulos D (2006) ltm: an R package for latent variable modeling and item response theory analyses. J Stat Software 17(5):

Saccone SF, Hinrichs AL, Saccone NL, Chase GA, Konvicka K, Madden PAF, Breslau N, Johnson EO, Hatsukami D, Pomerleau O, Swan GE, Goate AM, Rutter J, Bertelsen S, Fox L, Fugman D, Martin NG, Montgomery GW, Wang JC, Ballinger DG, Rice JP, Bierut LJ (2007) Cholinergic nicotinic receptor genes implicated in a nicotine dependence association study targeting 348 candidate genes with 3713 SNPs. Human Molecular Genetics 16(1):36-49

Saccone SF, Pergadia ML, Loukola A, Broms U, Montgomery GW, Wang JC, Agrawal A, Dick DM, Heath AC, Todorov AA, Maunu H, Heikkila K, Morley KI, Rice JP, Todd RD, Kaprio J, Peltonen L, Martin NG, Goate AM, Madden PAF (2007) Genetic linkage to chromosome 22q12 for a heavy-smoking quantitative trait in two independent samples. Am J Hum Genet 80(5):856-866

Slutske WS, Meier MH, Zhu G, Stratham DJ, Blaszczynski A, Martin NG (2009) The Australian twin study of gambling (OZ-GAM): Rationale, sample description, predictors of participation, and a first look at sources of individual differences in gambling involvement. Twin Research and Human Genetics 1263-78

Tellegen A (2000) Manual of the Multidimensional Personality Questionnaire. University of Minnesota Press, Minneapolis

Tellegen A, Waller NG (2008) Exploring personality through test construction: Development of the Multidimensional Personality Questionnaire. InThe SAGE handbook of personality theory and assessment, pp

Terracciano A, McCrae RR, Brant LJ, Costa PT (2005) Hierarchical linear modeling analyses of the NEO-PI-R scales in the Baltimore longitudinal study of aging. Psychology and Aging 20(3):493-506

van den Oord EJCG, Kuo PH, Hartmann AM, Webb BT, Moller HJ, Hettema JM, Giegling I, Bukszar J, Rujescu D (2008) Genomewide association analysis followed by a replication study implicates a novel candidate gene for neuroticism. Archives of General Psychiatry 65(9):1062-1071

Verhagen AJ, Fox JP (2012) Bayesian tests of measurement invariance. British J Math Stat Psychol 10.1111/j.2044-8317.2012.02059.x

Verhagen AJ, Fox JP (2013) Longitudinal measurement in health-related surveys. A Bayesian joint growth model for multivariate ordinal responses. Stat Med 32(17):2988-3005

Wilde GJS (1970) Neurotische Labiliteit Gemeten Volgens de Vragenlijstmethode (The Questionnaire Method as a Means of Measuring Neurotic Instability). Van Rossen, Amsterdam

Wright MJ, Martin NG (2004) Brisbane Adolescent Twin Study: Outline of study methods and research projects. Australian Journal of Psychology 56(2):65-78
